# Transition from depression-free to death in late life: characteristics of bidirectional transitions in depression symptoms

**DOI:** 10.1017/S2045796025100310

**Published:** 2025-12-01

**Authors:** Xinrui Cui, Guirong Song, Dongmei Hu, Guorong Li, Ying Zhang, Yanan Ma, Xiao Tang

**Affiliations:** 1 Department of Health Statistics, School of Public Health, Dalian Medical University, Dalian, Liaoning, China; 2 Department of Epidemiology and Health Statistics, School of Public Health, Capital Medical University, Beijing, China; 3 Key Laboratory of Environmental Stress and Chronic Disease Control & Prevention, Ministry of Education, China Medical University; Department of Biostatistics and Epidemiology, School of Public Health, China Medical University, Shenyang, Liaoning, China;; 4 Health Sciences Institute, China Medical University; Liaoning Key Laboratory of Obesity and Glucose/Lipid Associated Metabolic Diseases, China Medical University, Shenyang, Liaoning, China

**Keywords:** depressive symptoms, multi-state Markov model, older adults, state transition

## Abstract

**Aims:**

Depression among middle-aged and older adults is a critical public health priority. Clarifying the dynamic evolution of depression is essential for establishing prevention and intervention strategies; however, relevant research is limited. The aim of this study was to elucidate the transition patterns underlying different depressive symptoms (DS) states.

**Methods:**

Data from the China Health and Retirement Longitudinal Study were utilised in this study, which included participants aged ≥45 years with multiple DS assessments via the Center for Epidemiological Studies Depression Scale. Multi-state Markov models were employed to estimate transition probabilities and intensities between DS states, the total length of stay and mean sojourn time in each state and the hazard ratios (HRs) of factors.

**Results:**

Among 19,991 participants (average follow-up: 7.3 years), the 10-year cumulative probabilities of transition from non-DS to depressive states increased by 19.4% in males and 31.8% in females. Mild DS was the most unstable state, with the highest transition intensities (males: 1.029; females: 0.970) and shortest sojourn time (males: 0.959 years; females: 1.022 years). Sex and age strongly influenced depressive state transitions. Compared to participants without chronic disease, those with ≥3 chronic diseases had a higher risk of developing mild DS (HR = 1.685, 95% Confidence Interval [CI]: 1.530–1.856) and transitioning to death from both the non-DS (HR = 2.905, 95% CI: 2.293–3.681) and severe-DS (HR = 3.429, 95% CI: 1.290–9.112) states, but a lower likelihood of recovery from mild DS (HR = 0.821, 95% CI: 0.749–0.900) and severe DS (HR = 0.730, 95% CI: 0.630–0.847). Compared to no participation in social activities, frequent participation was associated with a lower risk of progression to the mild-DS state (HR = 0.851, 95% CI: 0.785–0.920) and a greater likelihood of recovery from severe DS (HR = 1.169, 95% CI: 1.034–1.322). Being underweight was associated with an increased risk of mild-DS onset (HR = 1.338, 95% CI: 1.129–1.587) and transitioning to death from both the non-DS and mild-DS states, compared with individuals of normal weight.

**Conclusions:**

Our study revealed a continuous population shift towards depressive states and identified the mild-DS state as a critical intervention state owing to its instability. In addition to sex and age, modifiable factors, including chronic disease conditions, social activity participation and weight status, significantly influenced DS-state transitions, offering actionable insights for precision prevention strategies.

## Introduction

Depression is a globally prevalent chronic disease associated with a decline in quality of life and numerous adverse health outcomes (Fleetwood *et al.*, 2025). Approximately 322 million people worldwide have suffered from depression, accounting for 4.4% of the global population (World Health Organization, 2017). Although depression impacts all age groups, it presents distinct challenges among middle-aged and older adults (Wu *et al.*, 2025), with its global incidence having increased by 28.4% over the past two decades (Hu *et al.*, 2022). Depression in this population arises from unique contributors, such as multiple chronic conditions and significant life transitions (Mosconi *et al.*, 2023; Wu *et al.*, 2025), which can lead to poorer physical health, accelerated cognitive decline, increased disability and higher mortality rates (Agustini *et al.*, 2022; Fleetwood *et al.*, 2025). The substantial medical burden imposed by depression underscores the critical need to address this issue.

The natural course of depression is a dynamic process, characterised by fluctuations between various states, including normal, mild and severe depression. Previous studies have revealed recurrence rates of approximately 25%–44% and remission rates of 13%–23% (Beekman *et al.*, 2002; Hybels *et al.*, 2016; Jeuring *et al.*, 2018), reflecting the heterogeneous trajectories of depression development among middle-aged and older adults. However, the natural history of late-life depression as a continuum of change across different severity states remains unclear (Wu *et al.*, 2024). To date, only one Chinese study has investigated dynamic depressive state changes in middle-aged and older adults. However, this study failed to reveal a link between depressive states and mortality, which is a critical outcome closely tied to depression progression. Understanding the patterns underlying transitions between depressive states and their relationship with mortality risk is vital for providing targeted prevention and intervention strategies.

Additionally, extensive research has focused on a range of factors that influence depression among older adults, primarily including sociodemographic characteristics, lifestyle factors, health status and genetic predispositions (Cao *et al.*, 2021; Wu *et al.*, 2025). For instance, factors such as female sex, lower education levels, chronic conditions, lack of social support and abnormal weight status have been associated with an increased risk of depression (Wu *et al.*, 2025). However, the contribution of these factors to transitions across the spectrum of depressive states is still unclear. Given the greater susceptibility to medication side effects and lower treatment efficacy in older adults, identifying factors associated with progression to more severe states or improvement to milder states is crucial for the development of risk stratification systems and targeted prevention strategies.

Recently, multi-state Markov (MSM) models have been applied to analyse the dynamic progression of chronic conditions (Wu *et al.*, 2022). As an extension of the Cox proportional hazards model, MSM models are capable of describing complex, multi-stage disease processes. By estimating transition intensities between disease states, predicting transition probabilities, assessing sojourn times and identifying associated factors, MSM models provide comprehensive insights into disease development. However, their application to depression remains limited (Wu *et al.*, 2024).

To fill this research gap, we employed the MSM model using data from a nationally representative Chinese survey to (1) characterise transitions between depressive states and progression to mortality, (2) identify the most critical intervention stage and (3) determine key factors associated with these transitions.

## Methods

### Study population and design

The data for this study were obtained from the China Health and Retirement Longitudinal Study (CHARLS), a nationally representative longitudinal survey targeting middle-aged and older individuals in China. Since 2011, the CHARLS has been conducted every 2 years. A multistage sampling strategy was employed in this survey, which covered 28 provinces, 150 counties or districts and 450 villages or urban communities across the country, resulting in a total of 17,708 community residents being enrolled at baseline. Further details regarding the CHARLS survey have been previously described (Zhao *et al.*, 2014).

A total of 25,882 participants from five CHARLS waves (2011, 2013, 2015, 2018 and 2020) were included in our study. Participants without any measurement of depressive symptoms (DS) or those with only one measurement across the five waves were excluded (*n* = 4,097). The first observation was identified as the baseline for individuals. Participants with missing age data or a baseline age of less than 45 years were excluded (*n* = 1,763). Ultimately, a total of 19,991 participants with at least two depressive state assessments were eligible for subsequent analysis. A detailed flow chart of the study population selection process is shown in Figure S1.

### States of DS and death

In the CHARLS, DS were assessed by the 10-item Center for Epidemiological Studies Depression Scale (CESD-10) (Chen and Mui, 2014), which evaluates depressed mood over the past week. The survey consisted of items such as ‘I had trouble keeping my mind on what I was doing’, ‘I was bothered by things that usually don’t bother me’ and ‘I felt depressed’. Each item was rated according to the frequency of occurrence (0 = rarely or never, 1 = not too often, 2 = sometimes or half the time and 3 = most of the time). The total CESD-10 score was calculated as the sum of these ratings (range: 0–30), with higher scores indicating higher levels of DS. Three DS states were defined as follows: a CESD-10 score <10 indicated no DS (non-DS), a score of 10–15 indicated mild DS (mild-DS), and a score of ≥16 indicated severe DS (severe-DS) (Marshall *et al.*, 2018).

Death was also included as a state in our analysis, and all-cause death information for participants was obtained through death registration records during the follow-up.

### Factors

A range of factors, including sex, age (45–54, 55–64 and ≥65 years), education level (below elementary, elementary or middle school and above), marital status (married, others), residential region (urban, rural), chronic disease conditions (none, 1–2 chronic diseases and ≥3 chronic diseases), social participation (no, non-regular and frequent) and weight status (underweight, normal weight, overweight and obesity), were selected based on existing literature (Fan *et al.*, 2021; Wu *et al.*, 2025). Further details are provided in the Supplementary Materials.

### MSM model

**Figure 1.**
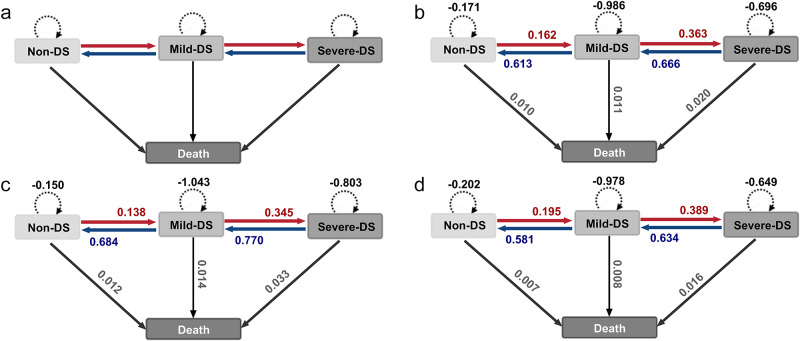
Structure of the multistate Markov model and estimated transition intensities. (a) Diagram of transitions between DS states in the multistate Markov model. Progression from non-DS to mild-DS and from mild-DS to severe-DS, recovery from mild-DS to non-DS and from severe-DS to mild-DS, as well as staying in the original state or dying in any state, were permitted. (b) Estimated transition intensities for all participants. (c) Estimated transition intensities for males. (d) Estimated transition intensities for females. DS: depressive symptoms.

A MSM model with four states was employed to describe the transition patterns of depression (Jackson, 2011). A detailed description of this model is provided in the Supplementary Materials. Non-DS, mild-DS and severe-DS were considered transient states, while death was defined as an absorbing state. Based on the time continuity of the model, we assumed that individuals can only transition between adjacent states instantaneously. In addition, individuals might stay in the original state or die in any state (Figure 1a).

The transition intensities, 10-year transition probabilities, mean sojourn time in each state, and total length of stay in different states over 10 years were estimated using an MSM model, which requires at least two assessments of disease states. Factors were all included in the model, with their effects on each transition assessed by hazard ratios (HR). In addition, we conducted stratified analyses based on various exposure factors and performed sensitivity analyses by excluding participants with mild-DS or severe-DS at baseline.

The MSM package in R 4.2.3 was used to construct the continuous MSM model.

## Results

### Baseline characteristics of participants

Baseline characteristics of participants are presented in Table 1. This study included 79,120 observations from 19,991 participants aged ≥45 years, comprising 49.2% males and 50.8% females, with an average follow-up period of 7.3 years. Among the participants, 57.8% of males and 56.1% of females were older than 55 years. Additionally, Table S1 presents baseline characteristics by DS state, and Table S2 details the observed transition frequencies between states.

**Table 1.** Baseline characteristics of participants

Factors	Males	Females
	(*n* = 9,844)	(*n* = 10,147)
Age (years)		
45–54	4,155 (42.2)	4,452 (43.9)
55–64	3,467 (35.2)	3,500 (34.5)
≥65	2,222 (22.6)	2,195 (21.6)
Education levels		
Below elementary	2,749 (27.9)	5,519 (54.4)
Elementary	3,153 (32.0)	2,220 (21.9)
Middle school and above	3,942 (40.0)	2,408 (23.7)
Marital status		
Married	9,061 (92.0)	8,753 (86.3)
Others	783 (8.0)	1,394 (13.7)
Residential regions		
Urban	3,959 (40.2)	4,282 (42.2)
Rural	5,885 (59.8)	5,865 (57.8)
Chronic disease conditions		
0	4,109 (41.7)	3,758 (37.0)
1–2	4,324 (43.9)	4,585 (45.2)
≥3	1,411 (14.3)	1,804 (17.8)
Social participation		
No	4,529 (46.0)	4,884 (48.1)
Non-regular	2,374 (24.1)	2,126 (21.0)
Frequent	2,941 (29.9)	3,137 (30.9)
Weight status		
Underweight	506 (5.1)	533 (5.3)
Normal	5,501 (55.9)	4,785 (47.2)
Overweight	2,918 (29.6)	3,399 (33.5)
Obesity	919 (9.3)	1,430 (14.1)
CESD-10 score	6.8 ± 5.7	8.7 ± 6.4

Values were *n* (percentages) or mean ± standard deviation.

CESD-10: the 10-item Center for Epidemiological Studies Depression Scale.

### Intensities of transitions between DS states

The mild-DS state had the highest transition intensities (males: 1.029; females: 0.970). Regarding deterioration, the transition intensity from the mild-DS state to the severe-DS state exceeded that from the non-DS state to the mild-DS state (males: 0.345 vs. 0.138; females: 0.389 vs. 0.195). Among recovery transitions, the transition intensity from the severe-DS to the mild-DS state was close to that from the mild-DS to the non-DS state (males: 0.770 vs. 0.684; females: 0.634 vs. 0.581). Additionally, the transition intensity from the non-DS, mild-DS and severe-DS states to death gradually increased (males: 0.012, 0.014, 0.033, respectively; females: 0.007, 0.008, 0.016, respectively) (Figure 1b–d and Table S3).

### Probabilities of transition between DS states

**Figure 2.**
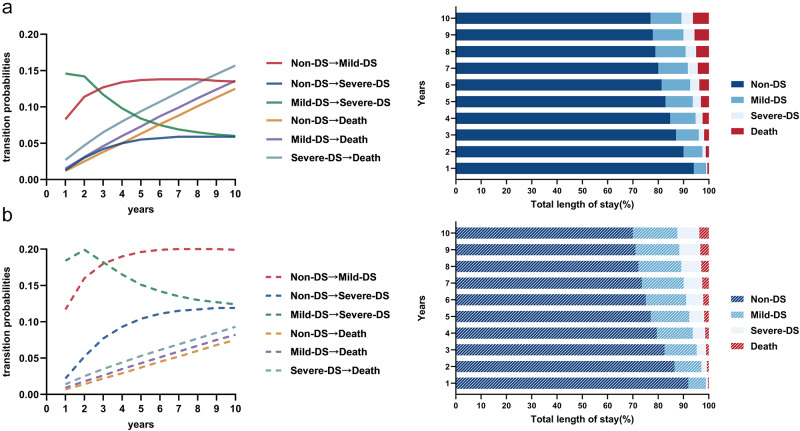
Transition probability curves and percentage of total length of stay over 10 years. (a) Probability curves of progression to depressive states and transition to death. (b) Percentage of total length of stay. Solid and dashed lines represent males and females, respectively. DS: depressive symptoms.

For progression, both males and females exhibited peak probabilities of transition from the mild-DS to severe-DS states during the initial 1–2 years, whereas the probabilities of transition from the non-DS to the mild-DS state progressively increased over time, becoming highest in years 3–9. Females had higher 10-year probabilities of progressing to both mild-DS (19.9% vs. 13.5%) and severe-DS (11.9% vs. 5.9%) states than males. Analysis of the probabilities of transition to death revealed clear severity dependence. Males showed 10-year probabilities of 12.5%, 13.6% and 15.7% for transitions from the non-DS, mild-DS and severe-DS states, respectively, while females exhibited lower but similarly graded probabilities (7.5%, 8.2% and 9.3%, respectively) (Figure 2a and Tables S4–S5). Additional transition patterns, including reversion and staying in an original state, are shown in Figure S2.

### Total length of stay and mean sojourn time in each DS state

As shown in Figure 2b and Table S6, the percentage of the total length of stay spent in the non-DS state over 10 years decreased (males: 94.0%–76.9%; females: 91.9%–70.0%), accompanied by proportional increases in the length of stay in the mild-DS (males: 4.9%–12.3%; females: 6.9%–17.6%) and severe-DS states (males: 0.5%–4.5%; females: 0.8%–8.7%). The mean sojourn time of individuals in the mild-DS state was shorter than that in the non-DS and severe-DS states (Table S7).

### Association of factors with transitions between DS states

The effects of factors on the transitions between different states are summarised in Table 2. Sex, age and chronic disease conditions were most significantly associated with progression, recovery and transition to death. Compared to males, females were more likely to progress to the mild-DS state from the non-DS state and were less likely to recover from depressive states. Conversely, females had lower risks of transitioning to death from the non-DS and severe-DS states than males. Compared with individuals aged 45–54 years, those aged 55–64 years exhibited a reduced risk of progression to mild DS (HR = 0.913, 95% CI: 0.841–0.992) but an elevated risk of transitioning to death from the non-DS state (HR = 2.368, 95% CI: 1.600–3.505). Those over 65 years also had a lower risk of developing mild DS; however, they had both a higher likelihood of recovery from depressive states and an increased risk of death from any DS state. A similar association was observed for each 1-year increment in age (Table S8).

**Table 2.** Hazard ratios of covariates associated with transitions between DS states

Factors	Deteriorate transition		Recovery transition		Death transition		
	Non-DS→mild-DS	Mild-DS→severe-DS	Mild-DS→non-DS	Severe-DS→mild-DS	Non-DS→death	Mild-DS→death	Severe-DS→death
Sex							
Males	1 (ref)	1 (ref)	1 (ref)	1 (ref)	1 (ref)	1 (ref)	1 (ref)
Females	**1.293 (1.203, 1.390)**	1.055 (0.932, 1.195)	**0.854 (0.797, 0.915)**	**0.846 (0.759, 0.942)**	**0.464 (0.384, 0.560)**	0.765 (0.423, 1.385)	**0.376 (0.262, 0.539)**
Age (years)							
45–54	1 (ref)	1 (ref)	1 (ref)	1 (ref)	1 (ref)	1 (ref)	1 (ref)
55–64	**0.913 (0.841, 0.992)**	0.946 (0.824, 1.086)	1.078 (0.995, 1.168)	0.969 (0.859, 1.095)	**2.368 (1.600, 3.505)**	1.774 (0.869, 3.625)	1.489 (0.698, 3.180)
≥65	**0.840 (0.764, 0.923)**	1.111 (0.943, 1.308)	**1.101 (1.007, 1.205)**	**1.215 (1.055, 1.400)**	**8.354 (5.803, 12.028)**	**4.026 (1.989, 8.150)**	**6.241 (3.178, 12.257)**
Education levels							
Below elementary	1 (ref)	1 (ref)	1 (ref)	1 (ref)	1 (ref)	1 (ref)	1 (ref)
Elementary	**0.788 (0.722, 0.860)**	**0.752 (0.650, 0.870)**	0.955 (0.878, 1.038)	0.993 (0.875, 1.125)	**0.777 (0.640, 0.943)**	0.444 (0.150, 1.316)	0.906 (0.605, 1.357)
Middle school and above	**0.629 (0.576, 0.687)**	**0.739 (0.635, 0.859)**	0.984 (0.906, 1.070)	1.100 (0.966, 1.253)	**0.615 (0.494, 0.765)**	1.026 (0.530, 1.989)	0.619 (0.336, 1.142)
Marital status							
Married	1 (ref)	1 (ref)	1 (ref)	1 (ref)	1 (ref)	1 (ref)	1 (ref)
Others	1.093 (0.982, 1.218)	0.940 (0.799, 1.106)	0.973 (0.885, 1.071)	**0.864 (0.758, 0.983)**	**2.293 (1.917, 2.742)**	1.182 (0.634, 2.204)	**2.056 (1.432, 2.953)**
Residential regions							
Urban	1 (ref)	1 (ref)	1 (ref)	1 (ref)	1 (ref)	1 (ref)	1 (ref)
Rural	**1.401 (1.303, 1.506)**	**1.157 (1.022, 1.310)**	0.995 (0.929, 1.065)	1.007 (0.904, 1.121)	1.036 (0.871, 1.233)	1.162 (0.635, 2.127)	0.928 (0.632, 1.363)
Chronic disease conditions							
0	1 (ref)	1 (ref)	1 (ref)	1 (ref)	1 (ref)	1 (ref)	1 (ref)
1–2	**1.283 (1.180, 1.396)**	1.064 (0.907, 1.248)	0.941 (0.866, 1.023)	0.923 (0.797, 1.069)	**1.622 (1.292, 2.037)**	1.520 (0.757, 3.051)	1.787 (0.678, 4.710)
≥3	**1.685 (1.530, 1.856)**	0.976 (0.826, 1.154)	**0.821 (0.749, 0.900)**	**0.730 (0.630, 0.847)**	**2.905 (2.293, 3.681)**	1.599 (0.773, 3.307)	**3.429 (1.290, 9.112)**
Social participation							
No	1 (ref)	1 (ref)	1 (ref)	1 (ref)	1 (ref)	1 (ref)	1 (ref)
Non-regular	0.920 (0.844, 1.003)	0.936 (0.812, 1.080)	**0.904 (0.833, 0.981)**	0.997 (0.881, 1.129)	**0.741 (0.597, 0.919)**	0.988 (0.211, 4.630)	**0.468 (0.258, 0.848)**
Frequent	**0.850 (0.785, 0.920)**	1.008 (0.877, 1.159)	0.929 (0.860, 1.002)	**1.169 (1.034, 1.322)**	**0.567 (0.438, 0.736)**	**4.294 (1.610, 11.452)**	0.116 (0.003, 4.605)
Weight status							
Normal	1 (ref)	1 (ref)	1 (ref)	1 (ref)	1 (ref)	1 (ref)	1 (ref)
Underweight	**1.338 (1.129, 1.587)**	1.068 (0.827, 1.378)	1.040 (0.886, 1.222)	1.014 (0.815, 1.262)	**1.758 (1.330, 2.322)**	**2.313 (1.205, 4.441)**	1.112 (0.559, 2.212)
Overweight	0.930 (0.862, 1.003)	1.033 (0.905, 1.180)	0.985 (0.916, 1.059)	**1.148 (1.023, 1.288)**	1.052 (0.880, 1.258)	0.356 (0.126, 1.008)	1.350 (0.911, 2.002)
Obesity	0.904 (0.811, 1.007)	1.011 (0.846, 1.208)	0.976 (0.879, 1.084)	1.071 (0.916, 1.254)	0.825 (0.585, 1.164)	0.582 (0.110, 3.093)	1.392 (0.787, 2.463)

Values were hazard ratios (95% CI).

Boldfaced data indicate statistical significance (*P* < 0.05).

DS: depressive symptoms.

Individuals with chronic comorbidities were more likely to develop mild DS than those without any chronic diseases, with HRs of 1.283 (95% CI: 1.180–1.396) and 1.685 (95% CI: 1.530–1.856) in the 1–2 and ≥3 chronic disease groups, respectively. In contrast, a diminished likelihood of recovering from the mild-DS and severe-DS states was found in the ≥3 chronic disease group. In addition, an increased risk of transitioning to death from DS states was observed in those with at least three chronic diseases.

Engaging frequently in social activities decreased the risk of developing mild DS (HR = 0.850, 95% CI: 0.785–0.920) and transitioning to death from the non-DS state (HR = 0.567, 95% CI: 0.438–0.736), while increasing the likelihood of recovery from the severe-DS state to the mild-DS state (HR = 1.169, 95% CI: 1.034–1.322). Non-regular participation in social activities was also associated with lower risks of transitioning to death from the non-DS state and severe-DS states. Additionally, underweight individuals had a higher likelihood of developing mild DS and were more prone to death from both the non-DS and mild-DS states compared with normal-weight individuals.

### Stratified analysis

**Figure 3.**
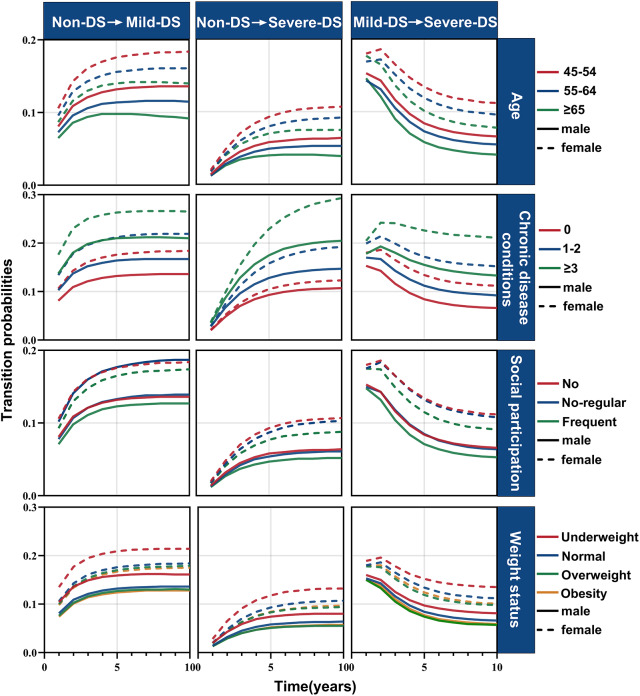
Transition probability curves of worsening to depressive states over 10 years, stratified by sex, age, chronic disease conditions, social participation and weight status. Model adjusted for age, education levels, residential regions, marital status, chronic disease conditions, social participation and weight status. DS: depressive symptoms.

The 10-year transition probabilities of progression to depressive states from sex- and factor-stratified analyses are presented in Figure 3 (Tables S9–S20). Females with ≥3 chronic diseases exhibited the highest probabilities of progression to depressive states. Higher probabilities of transitioning to the mild-DS and severe-DS states were also observed in participants who were young, lacked social participation and were underweight. The probabilities of transitioning to death, recovering from depressive states and remaining in their original states are shown in Figures S3–S5, respectively.

**Figure 4.**
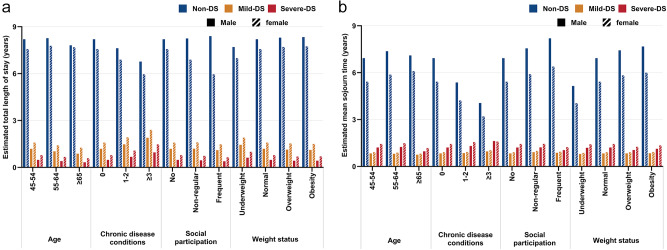
Total length of stay over 10 years and mean sojourn time, stratified by sex, age, chronic disease conditions, social participation and weight status. (a) Total length of stay in each state. (b) Mean sojourn time in each state. Model adjusted for age, education levels, residential regions, marital status, number of chronic diseases, social participation and weight status. DS: depressive symptoms.

The total length of stay over 10 years and the mean sojourn time by sex and other factors are presented in Figure 4 (Tables S21–S22). Individuals with at least three chronic diseases had the longest duration and mean sojourn time in the mild-DS and severe-DS states.

### Sensitivity analyses

Sensitivity analysis restricted to participants in the initial non-DS state revealed results consistent with the primary findings (Tables S23–S30 and Figures S6–S11). The mild-DS state showed the highest transition intensity and shortest mean sojourn time (Table S25 and Table S29). The transition probabilities of developing DS increased over 10 years, accompanied by progressive accumulation of total length of stay in depressive states (Figure S6 and Tables S26–S27). Sex, age and chronic disease condition remained the most significant factors associated with DS state transitions (Table S30).

## Discussion

In this study, the bidirectional transitions between DS states were clarified through an analysis of nationally representative longitudinal data, and several noteworthy findings were revealed. First, despite a relatively strong reversibility of DS states, individuals in the non-DS state still exhibited heightened probabilities of transitioning to both the mild-DS and severe-DS states over 10 years. This trend was accompanied by a decline in the total length of stay in the non-DS state and an increase in the time of maintained DS. Second, the mild-DS state emerged as a key intervention state owing to its instability, marked by the shortest duration and the highest transition intensities to the non-DS and severe-DS states. Third, sex, age and modifiable factors, including chronic disease conditions, social activity participation and weight status, were identified as critical contributors to DS state transitions.

Our findings revealed an increasing trend in the probability of transition from non-DS to depressive states over time, especially from non-DS to mild DS. Concurrently, the time spent in depressive states increased over time, illustrating the population’s progression towards depression. This finding is partially consistent with that of a national study in which data from the China Family Panel Study (CFPS) were evaluated; the study revealed increasing probabilities of transition from the non-DS state to the mild-DS and severe-DS states over 6 years, with 24.5% of the overall population experiencing this shift within this timeframe (Wu *et al.*, 2024). Findings from both studies suggested a significant onset of mild DS in the Chinese population. However, the estimated percentage of participants deteriorating to the severe-DS state from the non-DS state was greater in our study than in the CFPS. This discrepancy may be attributable to differences in sample size and analytical perspective. Although research on the dynamic changes in depression status remains limited, a rapid increase in the prevalence of depression among the Chinese population has been consistently reported (Ren *et al.*, 2020). Therefore, depression prevention strategies in middle-aged and older adults are essential.

Our study revealed, for the first time, that the mild-DS state is a notable state in the natural development of depression. The mild-DS state exhibited the shortest duration and had higher intensities of both worsening to the severe-DS state and recovering to the non-DS states, thereby demonstrating the unstable nature of the mild-DS state. These findings highlight that the mild-DS state is a key stage for intervention. In contrast, a critical intervention period was not identified in the CFPS, but its results emphasised that individuals had the shortest duration of severe DS (Wu *et al.*, 2024), which is inconsistent with our results. However, our findings were strongly supported by clinical observations. Individuals in the severe-DS state typically present with substantial symptom burden and functional impairment, often requiring pharmacological treatment or even combined interventions (Feifel *et al.*, 2017; Sabesan *et al.*, 2015). In contrast, individuals in the mild-DS state are characterised by a persistent low mood, along with alterations in sleep, appetite and cognitive-affective biases. These symptoms can often be ameliorated through psychological interventions, such as cognitive behavioural therapy, resulting in a relatively more favourable recovery trajectory (Du *et al.*, 2016). Notably, we further revealed that the speed of deterioration in the mild-DS state was faster than the mild-DS onset. Therefore, the findings from our study emphasise the importance of timely screening and intervention at the early stage to prevent the rapid deterioration of depression.

In the present study, sex was significantly associated with DS occurrence and recovery. Compared with males, females were more prone to developing DS and less likely to recover from depressive states. Our findings align with those of previous studies, which revealed a greater incidence of depression in females (Liu *et al.*, 2021), likely owing to their longer lifespans, higher widowhood rates, greater feelings of loneliness and increased caregiving responsibilities (Turner *et al.*, 2004). However, males were more likely to transition from the severe-DS state to death, consistent with prior research showing higher mortality rates among depressed males (Cuijpers *et al.*, 2014). The hypothesis of ‘sex differences in self-reported symptoms of depression’ has been proposed to explain this discrepancy (Shi *et al.*, 2021). Sex differences in coping with negative emotions and DS phenotypes, such as undiagnosed atypical DS in males, could also contribute to this phenomenon (Shi *et al.*, 2021). Consequently, both improving mental healthcare for females and encouraging emotional expression and accurate diagnosis in males are critical.

Our study also revealed that individuals over 55 years, particularly those over 65, were less likely to develop mild DS and more likely to recover from depressive states; however, they also had a higher risk of death. Furthermore, their mean sojourn times in both mild-DS and severe-DS states were shorter than those of individuals aged 45–54 years. Research on the role of age in DS state transition is limited; however, the association between DS prevalence and age has been explored in several studies (Fan *et al.*, 2021; Sutin *et al.*, 2013). For example, a study conducted in China revealed a decreased risk of DS with increasing age among adults over 45 years (Fan *et al.*, 2021). Middle-aged individuals typically experience greater familial, career and financial pressures than retired older individuals (Yang and D’Arcy, 2023), highlighting the need for additional psychological support for this population to prevent DS. Furthermore, this study observed an increased mortality risk in the non-DS state with advancing age, which may be attributed to age-related declines in physiological reserve, multimorbidity and immunosenescence (Cai *et al.*, 2022). Critically, for individuals in depressive states, the risk of death was significantly elevated only in those over 65 years. This suggests a synergistic effect between depression and advanced age, exacerbating frailty and depleting physiological reserve (Lee *et al.*, 2022). Hence, effective emotional management is imperative for older adults.

The current study also revealed a significant role for chronic disease conditions in the bidirectional transitions between DS states in middle-aged and older adults. The probability of progression to depressive states escalated with the number of chronic diseases. Moreover, those afflicted with chronic diseases tended to have a prolonged mean sojourn time in depressive states. Previous studies have shown a significant correlation between chronic diseases and incident depression. A meta-analysis of 40 studies revealed that individuals with multiple chronic diseases had twice the risk of developing depression as those with a single chronic disease and three times the risk of those without any chronic diseases (Read *et al.*, 2017). Nevertheless, the effect of chronic disease on dynamic changes in DS has been explored only in the CFPS, which revealed an association of multiple chronic diseases with elevated risks of DS onset and worsening. However, that study also revealed that the presence of a single disease had an apparent protective effect against the progression from mild to severe DS, which completely contrasts with our findings (Wu *et al.*, 2024). The detrimental effects of chronic diseases on depression can be explained through several pathways: the substantial burden and disability from multiple conditions (Verhaak *et al.*, 2014); shared immune-inflammatory mechanisms in diseases like hypertension and arthritis (Won and Kim, 2016); and overlapping neuroplasticity changes from long-term pain and depression (Ru *et al.*, 2022). Our study also found that chronic diseases increased the risk of transitioning to death from non-DS or severe-DS states. In individuals with non-DS, even 1–2 diseases significantly increased risk, supporting the direct physiological burden of comorbidities (Fan *et al.*, 2022). However, among individuals with severe DS, only those with ≥3 chronic diseases exhibited a sharp rise in mortality risk. This threshold effect suggests severe depression synergises with high multimorbidity to overwhelm physiological resilience, resulting in an escalation of vulnerability (Prigge et al., 2022). Consequently, managing chronic diseases in older adults is crucial for preventing depression and lowering mortality.

This study revealed that social participation protected against DS onset and aided severe-DS recovery, consistent with prior research (Xiao *et al.*, 2021). Such activities improve social connectedness, increase happiness and provide support systems and positive outlooks while reducing negative emotions, thereby improving mental health in older adults (Tengku Mohd *et al.*, 2019). Compared with normal-weight individuals, underweight individuals were more susceptible to developing mild DS, whereas those with overweight/obesity did not exhibit this trend. This disparity may involve biological mechanisms. A lower BMI reduces leptin (an antidepressant hormone from adipose tissue), potentially triggering DS (Lawson *et al.*, 2012). Additionally, weight-related illnesses and poor health may contribute to depression (Stubbs *et al.*, 2017). Underweight individuals also had an increased mortality risk in both the non-DS and mild DS states, possibly due to impaired immune-endocrine function and multi-system fragility associated with a low BMI (Global *et al.*, 2016). These results highlight the need for targeted mental health interventions for underweight middle-aged and older adults, emphasising social participation and weight maintenance.

## Strengths and limitations

Our study makes three primary contributions. First, it is a pioneering study exploring the dynamic evolution of depression and transitions from DS states to death in a large national population. Second, we identified the mild-DS state as a critical state for depression intervention, underscoring the importance of timely screening and management. Third, we provided a comprehensive assessment of factors influencing DS state transitions, with a detailed characterisation of depression progression across different groups.

Several limitations warrant consideration. First, measuring depression with a scale rather than a clinical diagnosis may have caused misclassification bias in the categorisation of DS states. Second, to ensure model robustness, some potential risk factors, such as physical activity and income, were excluded owing to substantial missing data, which may have led to potential residual confounding. Third, owing to the lack of cause-specific death data, we addressed only all-cause death in our analysis. Fourth, healthcare interventions during follow-up may have altered the natural course of the disease, leading to increased odds of disease reversal. Fifth, while our model accurately predicted the intensity and probability of transitions between DS states, it was unable to predict the exact timing of depression occurrence.

## Conclusion

Among middle-aged and older adults, our findings indicate a trend of progression toward depressive states over 10 years. The mild-DS state represents an unstable state in the natural development of depression, serving as a critical window for intervention. Females, middle-aged individuals and those with chronic comorbidities, low social participation and underweight status warrant special attention in depression intervention strategies.

## Supporting information

10.1017/S2045796025100310.sm001 Cui et al. supplementary material Cui et al. supplementary material

## Data Availability

The data supporting this study can be accessed at: https://charls.pku.edu.cn/.

## Supplementary material

The supplementary material for this article can be found at https://doi.org/10.1017/S2045796025100310.
