# Supplementary material for: Transition from depression-free to death in late life: characteristics of bidirectional transitions in depression symptoms
Source: Epidemiol Psychiatr Sci. 2025 Dec 1;34:e56. doi: 10.1017/S2045796025100310 (PMC12722190; doi:10.1017/S2045796025100310)
Supplement: Cui et al. supplementary material [file S2045796025100310sup001.docx]

**Figure S1** Flow chart of the selection of the study population

**Supplementary Note:** Definition of covariates

**Supplementary Note:** Multi-state Markov model

**Table S1** Baseline characteristics of participants stratified by their DS states

**Table S2** Observed frequencies of transitions between DS states over the follow-up period

**Table S3** Transition intensities estimated using the multistate model

**Table S4** Estimated transition probabilities over 10 years in males

**Table S5** Estimated transition probabilities over 10 years in females

**Figure S2** **Transition probability curves.** (A) transition probability curves of reversion; (B) transition probability curves of staying in an original state. Solid and dashed line represent males and females, respectively. DS: depressive symptoms.

**Table S6** Percentage of total length of stay in each DS state over 10 years

**Table S7** Estimated mean sojourn time in each DS state

**Table S8**. Hazard ratios of covariates associated with transitions between DS states (age modeled as a continuous variable)

**Table S9** Estimated probabilities of staying in non-DS over 10 years, stratified by sex and other factors

**Table S10** Estimated transition probabilities from non-DS to mild-DS over 10 years, stratified by sex and other factors

**Table S11** Estimated transition probabilities from non-DS to severe-DS over 10 years, stratified by sex and other factors

**Table S12** Estimated transition probabilities from non-DS to death over 10 years, stratified by sex and other factors

**Table S13** Estimated transition probabilities from mild-DS to non-DS over 10 years, stratified by sex and other factors

**Table S14** Estimated probabilities of staying in mild-DS over 10 years, stratified by sex and other factors

**Table S15** Estimated transition probabilities from mild-DS to severe-DS over 10 years, stratified by sex and other factors

**Table S16** Estimated transition probabilities from mild-DS to death over 10 years, stratified by sex and other factors

**Table S17** Estimated transition probabilities from severe-DS to non-DS over 10 years, stratified by sex and other factors

**Table S18** Estimated transition probabilities from severe-DS to mild-DS over 10 years, stratified by sex and other factors

**Table S19** Estimated probabilities of staying in severe-DS over 10 years, stratified by sex and other factors

**Table S20** Estimated transition probabilities from severe-DS to death over 10 years, stratified by sex and other factors

**Figure S3 Probability curves of t****ransitioning to death over 10 years, stratified by sex, age, chronic diseases conditions, social participation and weight status.** Model adjusted for age, education levels, residential regions, marital status, number of chronic diseases, social participation, and weight status. DS: depressive symptoms.

**Figure S4 Probability curves of recovering over 10 years, stratified by sex, age, chronic diseases conditions, social participation and weight status.** Model adjusted for age, education levels, residential regions, marital status, number of chronic diseases, social participation, and weight status. DS: depressive symptoms.

**Figure S5 Probability curves of staying in original states over 10 years, stratified by sex, age, chronic diseases conditions, social participation and weight status.** Model adjusted for age, education levels, residential regions, marital status, number of chronic diseases, social participation, and weight status. DS: depressive symptoms.

**Table** **S21** Estimated total length of stay in 10 years stratified by sex and other factors

**Table S22** Estimated mean sojourn time stratified by sex and other factors

**Table S23** Baseline characteristics of participants in the sensitivity analysis

**Table S24** Observed frequencies of transitions between DS states in the sensitivity analysis

**Table S25** Estimated transition intensities in the sensitivity analysis

**Table S26** Estimated transition probabilities over 10 years for males in the sensitivity analysis

**Table S27** Estimated transition probabilities over 10 years for females in the sensitivity analysis

**Table S28** Percentage of total length of stay over 10 years in the sensitivity analysis

**Table S29** Estimated mean sojourn time in each DS state from the sensitivity analysis

**Table S30** Hazard ratios of covariates associated with transitions between DS states in the sensitivity analysis

**Figure S6** **Transition probability curves and percentage of total length of stay over 10 years in the sensitivity analysis** (A) probability curves of progression to depressive states and transition to death; (B) probability curves of reversion; (C)probability curves of staying in an original state; (D) percentage of total length of stay. DS: depressive symptoms.

**Figure S7 Probability curves of worsening to depressive states over 10 years in the sensitivity analysis, stratified by sex, age, chronic diseases conditions, social participation and weight status.** Model adjusted for age, education levels, residential regions, marital status, number of chronic diseases, social participation, and weight status. DS: depressive symptoms.

**Figure S8 Probability curves of transitioning to death over 10 years in the sensitivity analysis, stratified by sex, age, chronic diseases conditions, social participation and weight status.** Model adjusted for age, education levels, residential regions, marital status, number of chronic diseases, social participation, and weight status. DS: depressive symptoms.

**Figure S9** **Probability curves of recovering over 10 years** **in the sensitivity analysis, stratified by sex, age, chronic diseases conditions, social participation and weight status.** Model adjusted for age, education levels, residential regions, marital status, number of chronic diseases, social participation, and weight status. DS: depressive symptoms.

**Figure S10 Probability curves of staying in original states over 10 years in the sensitivity analysis, stratified by sex, age, chronic diseases conditions, social participation and weight status.** Model adjusted for age, education levels, residential regions, marital status, number of chronic diseases, social participation, and weight status. DS: depressive symptoms.

**Figure S11 Total length of stay in 10 years and mean sojourn time in the sensitivity analysis, stratified by sex, age, chronic diseases conditions, social participation and weight status**. (A) total length of stay in each state; (B)mean sojourn time in each state. Model adjusted for age, education levels, residential regions, marital status, number of chronic diseases, social participation, and weight status. DS: depressive symptoms.

**
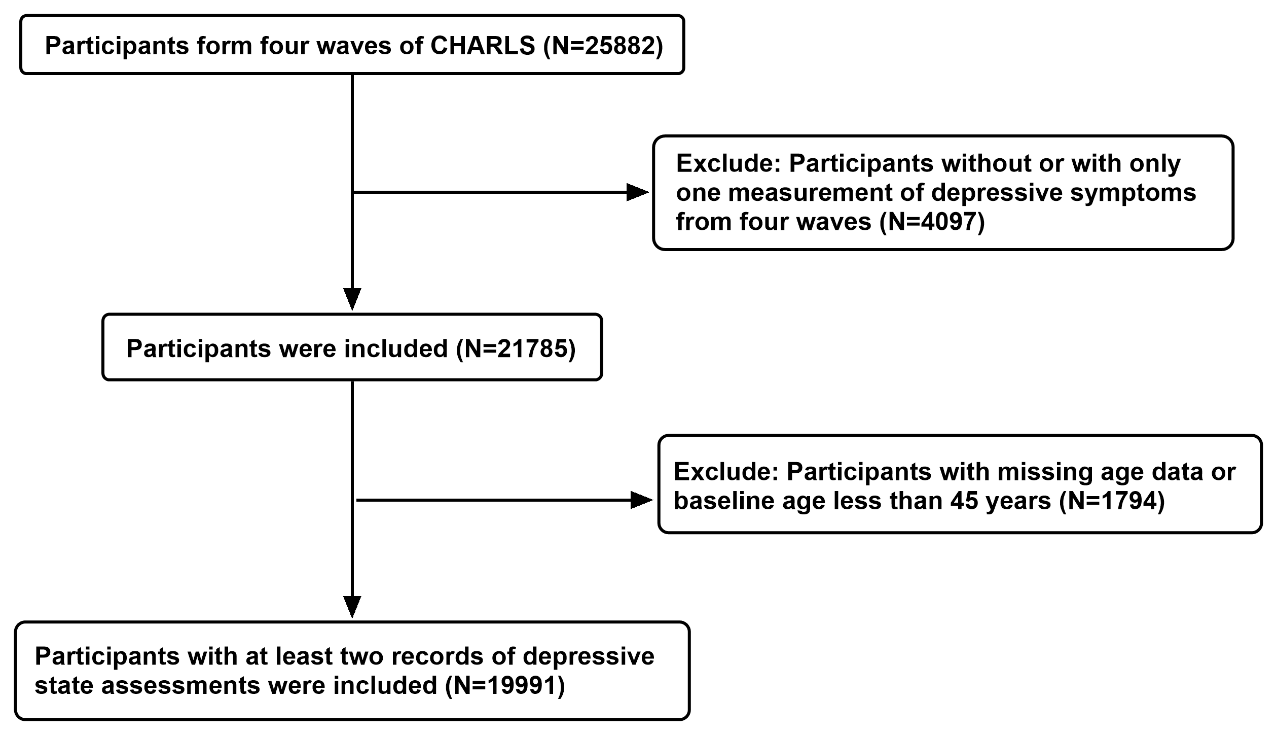
**

**Figure S1** Flow chart of the selection of the study population

**Supplementary Note: Definition of covariates**

A range of factors, including sex, age (45–54, 55–64, and ≥65 years), education level (below elementary, elementary or middle school and above), marital status (married, others), residential region (urban, rural), chronic disease conditions (none, 1–2 chronic diseases, and ≥3 chronic diseases), social participation (no, non-regular, and frequent), and weight status (underweight, normal weight, overweight, and obesity), were selected. Chronic disease conditions, including hypertension, diabetes, cancer, lung disease, heart problems, stroke, psychological problems, arthritis, dyslipidaemia, liver disease, kidney disease, stomach or digestive system diseases, asthma, and memory problems, were self-reported by the participants. Social participation was measured based on self-reported involvement in eight types of activities. These included: interacting with friends; playing Ma-Jong or cards or going to a community club; providing help to people who live elsewhere; going to a sport or other kinds of club; participating in community organizations; doing voluntary work or caring for a sick or disabled adult living outside the household; ,attending an educational or training course, engaging in other activities. The frequency of each activity was rated (0=never, 1=not regularly, 2=almost every week, and 3= almost daily). A total social activity score was calculated (range: 0–24) and categorized into three levels: ≥3 indicated frequent participation, 1–2 indicated non-regular participation, and 0 indicated no participation (Li et al., 2020). Weight and height were measured using standard instruments. Body mass index (BMI) was calculated as weight (kilogram) divided by height (meter) squared. Participants were categorised as underweight (< 18.5 kg/m2), normal weight (18.5–23.9 kg/m2), overweight (24.0–27.9 kg/m2), or obesity (≥28.0 kg/m2) based on Chinese BMI criteria.

**Supplementary Note:** Multi-state Markov model

The Markov model depicts the transition speed of a population between different states by the transition intensity. *X (t)=r* indicates that the individual is in state *r* at time *t*, and the intensity ($q_{rs}$) of the individual's transition from state *r* to state *s* within the interval (*t, t+Δt*) is:

$$q_{rs}(t)=\lim_{\Delta t\to0}\frac{P\left( X\left( t+\Delta t \right)=s \right|X\left( t \right)=r)}{\Delta t}$$

In the current study, non-DS, mild DS, severe DS, and death were labeled as states 1, 2, 3, and 4 respectively. Only the transition intensities (q) between adjacent state (as connected by arrows in Figure 1) were assigned non-zero values; all others were set to zero. The corresponding transition intensity matrix Q was defined as follows:

$$Q=\left[ \begin{matrix} -(q_{12}+q_{14}) & q_{12} & 0 & q_{14} \\ q_{21} & -(q_{21}+q_{23}+q_{24}) & q_{23} & q_{24} \\ 0 & q_{32} & -(q_{32}+q_{34}) & q_{34} \\ 0 & 0 & 0 & 0 \end{matrix} \right]$$

The multi-state Markov model can evaluate the impact of different covariates on the transition intensity ($q_{rs}$):

$q_{rs}=q_{rs}^{0}$*$e^{（\beta_{1}x_{1}+\beta_{2}x_{2}+\beta_{3}x_{3}\ldots+\beta_{k}x_{k}）}$

$q_{rs}^{0}$ represents the baseline transition intensity from state *r* to state *s* when all covariates are set to 0. $x_{i}$（i=1, 2……k）represents the *i*-th covariate, and $\beta_{i}$ is the regression coefficient corresponding to covariate $x_{i}$. Therefore, exp ($\beta_{i}$) is the hazard ratio (HR), quantifying the effect of each unit change in the covariate.

The transition intensity indicates a population’s speed of transition between different state. The transition probability reflects the likelihood of a transition between different states occurring within a given time frame. The mean sojourn time represents the average period of a single stay in a state before transitioning. The total length of stay estimates the cumulative time spent in a state between two future times.

**Table S1** Baseline characteristics of participants stratified by their DS states

| **Factors** | **Non-DS**  (N=13149) | **Mild-DS**  (N=4227) | **Severe-DS**  (N=2524) |
| --- | --- | --- | --- |
| **Sex** |  |  |  |
| Male | 7149 (54.4) | 1785 (42.2) | 910 (34.8) |
| Female | 6000 (45.6) | 2442 (57.8) | 1705 (65.2) |
| **Age (years)** |  |  |  |
| 45-54 | 6046 (46.0) | 1659 (39.2) | 902 (34.5) |
| 55-64 | 4407 (33.5) | 1534 (36.3) | 1026 (39.2) |
| ≥65 | 2696 (20.5) | 1034 (24.5) | 687 (26.3) |
| **Education levels** |  |  |  |
| Elementary below | 4665 (35.5) | 2053 (48.6) | 1550 (59.3) |
| Elementary | 3664 (27.9) | 1119 (26.5) | 590 (22.6) |
| Middle school and above | 4820 (36.7) | 1055 (25.0) | 475 (18.2) |
| **Residential regions** |  |  |  |
| Urban | 5952 (45.3) | 1554 (36.8) | 735 (28.1) |
| Rural | 7197 (54.7) | 2673 (63.2) | 1880 (71.9) |
| **Marital status** |  |  |  |
| Married | 12027 (91.5) | 3658 (86.5) | 2129 (81.4) |
| Other | 1122 (8.5) | 569 (113.5) | 486 (18.6) |
| **Chronic disease conditions** |  |  |  |
| 0 | 5951 (45.3) | 1312 (31.0) | 604 (23.1) |
| 1-2 | 5683 (43.2) | 2006 (47.5) | 1220 (46.7) |
| ≥3 | 1515 (11.5) | 909 (21.5) | 791 (30.2) |
| **Social participation** |  |  |  |
| No | 5774 (43.9) | 2164 (51.2) | 1475 (56.4) |
| Non-regular | 2962 (22.5) | 974 (23.0) | 564 (21.6) |
| Frequent | 4413 (33.6) | 1089 (25.8) | 576 (22.0) |
| **Weight status** |  |  |  |
| Underweight | 552 (4.2) | 270 (6.4) | 217 (8.3) |
| Normal | 6610 (50.3) | 2257 (53.4) | 1419 (54.3) |
| Overweight | 4375 (33.3) | 1231(29.1) | 711 (27.2) |
| Obesity | 1612 (12.3) | 469 (11.1) | 268 (10.2) |
| **CESD-10 score** | 4.2±2.8 | 12.1±1.7 | 19.8±3.4 |

Values were n (percentages) or mean ± standard deviation**.**

CESD-10: the 10-item Center for Epidemiological Studies Depression Scale.

**Table S2** Observed frequencies of transitions between DS states over the follow-up period

| **Original state** | **Follow-up state** | | | |
| --- | --- | --- | --- | --- |
|  | **Non-DS** | **Mild-DS** | **Severe-DS** | **Death** |
| **Overall** |  |  |  |  |
| Non-DS | 30677 (79.0) | 5328 (13.7) | 1850 (4.8) | 959 (2.5) |
| Mild-DS | 6593 (53.5) | 3457 (28.1) | 1923 (15.6) | 346 (2.8) |
| Severe-DS | 2571 (32.2) | 2488 (31.1) | 2630 (32.9) | 307 (3.8) |
| **Males** |  |  |  |  |
| Non-DS | 17166 (81.9) | 2406 (11.5) | 756 (3.6) | 644 (3.1) |
| Mild-DS | 3043 (58.8) | 1327 (25.6) | 612 (11.8) | 195 (3.8) |
| Severe-DS | 1007 (37.7) | 797 (29.9) | 717 (26.9) | 148 (5.6) |
| **Females** |  |  |  |  |
| Non-DS | 13510 (75.7) | 2922 (16.4) | 1094 (6.1) | 315 (1.8) |
| Mild-DS | 3550 (49.7) | 2130 (29.8) | 1311 (18.4) | 151 (2.1) |
| Severe-DS | 1564 (29.4) | 1691 (31.7) | 1913 (35.9) | 159 (3.00) |

Values were n (percentages).

DS: depressive symptoms.

**Table S3** Transition intensities estimated using the multistate model

| **Original state** | **Follow-up state** | | | |
| --- | --- | --- | --- | --- |
|  | **Non-DS** | **Mild-DS** | **Severe-DS** | **Death** |
| **Overall** |  |  |  |  |
| Non-DS | -0.171  (-0.177, -0.66) | 0.162  (0.157,0.167) | 0 | 0.010  (0.009,0.011) |
| Mild-DS | 0.613  (0.596,0.632) | -0.986  (-1.013, -0.960) | 0.363  (0.344,0.382) | 0.011  (0.007,0.015) |
| Severe-DS | 0 | 0.666  (0.636,0.696) | -0.696  (-0.717, -0.656) | 0.020  (0.016,0.025) |
| **Males** |  |  |  |  |
| Non-DS | -0.150  (-0.157, -0.144) | 0.138  (0.132,0.145) | 0 | 0.012  (0.011,0.014) |
| Mild-DS | 0.684  (0.653,0.716) | -1.043  (-1.088, -1.000) | 0.345  (0.315,0.377) | 0.014  (0.009,0.024) |
| Severe-DS | 0 | 0.770  (0.712,0.833) | -0.803  (-0.866, -0.744) | 0.033  (0.024,0.044) |
| **Females** |  |  |  |  |
| Non-DS | -0.202  (-0.211, -0.194) | 0.195  (0.187,0.204) | 0 | 0.007  (0.006,0.008) |
| Mild-DS | 0.581  (0.558,0.605) | -0.978  (-1.013, -0.944) | 0.389  (0.364,0.416) | 0.008  (0.005,0.014) |
| Severe-DS | 0 | 0.634  (0.599,0.671) | -0.649  (-0.687, -0.614) | 0.016  (0.012,0.021) |

Values were intensity (95%CI).

DS: depressive symptoms.

**Table S4** Estimated transition probabilities over 10 years in males

| **Years** | **Non-DS→**  **Non-DS** | **Non-DS→**  **Mild-DS** | **Non-DS→**  **Severe-DS** | **Non-DS→**  **Death** | **Mild-DS→**  **Non-DS** | **Mild-DS→**  **Mild-DS** |
| --- | --- | --- | --- | --- | --- | --- |
| Year 1 | 0.892 | 0.083 | 0.013 | 0.012 | 0.410 | 0.428 |
| Year 2 | 0.831 | 0.114 | 0.030 | 0.025 | 0.562 | 0.265 |
| Year 3 | 0.793 | 0.127 | 0.042 | 0.038 | 0.630 | 0.206 |
| Year 4 | 0.765 | 0.134 | 0.050 | 0.050 | 0.663 | 0.179 |
| Year 5 | 0.745 | 0.137 | 0.055 | 0.063 | 0.679 | 0.164 |
| Year 6 | 0.728 | 0.138 | 0.057 | 0.076 | 0.685 | 0.154 |
| Year 7 | 0.715 | 0.138 | 0.059 | 0.088 | 0.684 | 0.147 |
| Year 8 | 0.702 | 0.138 | 0.059 | 0.101 | 0.681 | 0.142 |
| Year 9 | 0.691 | 0.136 | 0.059 | 0.113 | 0.675 | 0.139 |
| Year 10 | 0.681 | 0.135 | 0.059 | 0.125 | 0.668 | 0.136 |

**Table S4 (cont.)** Estimated transition probabilities over 10 years in males

| **Years** | **Mild-DS→**  **Severe-DS** | **Mild-DS→**  **Death** | **Severe-DS→**  **Non-DS** | **Severe-DS→**  **Mild-DS** | **Severe-DS→**  **Severe-DS** | **Severe-DS→**  **Death** |
| --- | --- | --- | --- | --- | --- | --- |
| Year 1 | 0.146 | 0.015 | 0.142 | 0.326 | 0.505 | 0.027 |
| Year 2 | 0.142 | 0.031 | 0.332 | 0.316 | 0.304 | 0.047 |
| Year 3 | 0.117 | 0.046 | 0.469 | 0.262 | 0.204 | 0.065 |
| Year 4 | 0.098 | 0.060 | 0.555 | 0.218 | 0.147 | 0.080 |
| Year 5 | 0.084 | 0.073 | 0.605 | 0.187 | 0.113 | 0.094 |
| Year 6 | 0.075 | 0.087 | 0.633 | 0.167 | 0.092 | 0.107 |
| Year 7 | 0.069 | 0.099 | 0.646 | 0.154 | 0.079 | 0.120 |
| Year 8 | 0.065 | 0.112 | 0.651 | 0.146 | 0.071 | 0.133 |
| Year 9 | 0.062 | 0.124 | 0.650 | 0.139 | 0.065 | 0.145 |
| Year 10 | 0.060 | 0.136 | 0.647 | 0.135 | 0.062 | 0.157 |

DS: depressive symptoms.

**Table S5** Estimated transition probabilities over 10 years in females

| **Years** | **Non-DS→**  **Non-DS** | **Non-DS→**  **Mild-DS** | **Non-DS→**  **Severe-DS** | **Non-DS→**  **Death** | **Mild-DS→**  **Non-DS** | **Mild-DS→**  **Mild-DS** |
| --- | --- | --- | --- | --- | --- | --- |
| Year 1 | 0.854 | 0.117 | 0.022 | 0.007 | 0.349 | 0.459 |
| Year 2 | 0.773 | 0.160 | 0.052 | 0.014 | 0.477 | 0.306 |
| Year 3 | 0.722 | 0.180 | 0.077 | 0.022 | 0.535 | 0.256 |
| Year 4 | 0.687 | 0.190 | 0.093 | 0.029 | 0.565 | 0.235 |
| Year 5 | 0.663 | 0.196 | 0.104 | 0.037 | 0.582 | 0.223 |
| Year 6 | 0.646 | 0.199 | 0.111 | 0.045 | 0.591 | 0.216 |
| Year 7 | 0.633 | 0.200 | 0.115 | 0.052 | 0.595 | 0.211 |
| Year 8 | 0.622 | 0.200 | 0.117 | 0.060 | 0.596 | 0.207 |
| Year 9 | 0.614 | 0.200 | 0.119 | 0.068 | 0.595 | 0.204 |
| Year 10 | 0.606 | 0.199 | 0.119 | 0.075 | 0.593 | 0.201 |

**Table S5 (cont.)** Estimated transition probabilities over 10 years in females

| **Years** | **Mild-DS→**  **Severe-DS** | **Mild-DS→**  **Death** | **Severe-DS→**  **Non-DS** | **Severe-DS→**  **Mild-DS** | **Severe-DS→**  **Severe-DS** | **Severe-DS→**  **Death** |
| --- | --- | --- | --- | --- | --- | --- |
| Year 1 | 0.184 | 0.009 | 0.104 | 0.300 | 0.582 | 0.014 |
| Year 2 | 0.199 | 0.018 | 0.254 | 0.324 | 0.396 | 0.025 |
| Year 3 | 0.182 | 0.026 | 0.372 | 0.297 | 0.296 | 0.035 |
| Year 4 | 0.165 | 0.035 | 0.452 | 0.269 | 0.235 | 0.044 |
| Year 5 | 0.151 | 0.043 | 0.504 | 0.247 | 0.196 | 0.053 |
| Year 6 | 0.142 | 0.051 | 0.537 | 0.231 | 0.170 | 0.061 |
| Year 7 | 0.135 | 0.059 | 0.557 | 0.220 | 0.153 | 0.069 |
| Year 8 | 0.130 | 0.067 | 0.569 | 0.212 | 0.142 | 0.077 |
| Year 9 | 0.127 | 0.075 | 0.575 | 0.207 | 0.134 | 0.085 |
| Year 10 | 0.124 | 0.082 | 0.577 | 0.202 | 0.128 | 0.093 |

DS: depressive symptoms.





**Figure S2** **Transition probability curves.** (A) transition probability curves of reversion; (B) transition probability curves of staying in an original state. Solid and dashed line represent males and females, respectively. DS: depressive symptoms.

**Table S6** Percentage of total length of stay in each DS state over 10 years

| **Years** | **Males (%)** | | | | **Females (%)** | | | |
| --- | --- | --- | --- | --- | --- | --- | --- | --- |
|  | **Non-DS** | **Mild-DS** | **Severe-DS** | **Death** | **Non-DS** | **Mild-DS** | **Severe-DS** | **Death** |
| Year 1 | 94.0 | 4.9 | 0.5 | 0.6 | 91.9 | 6.9 | 0.8 | 0.3 |
| Year 2 | 90.0 | 7.5 | 1.3 | 1.2 | 86.5 | 10.6 | 2.3 | 0.7 |
| Year 3 | 87.0 | 9.0 | 2.1 | 1.9 | 82.5 | 12.8 | 3.7 | 1.1 |
| Year 4 | 84.7 | 10.1 | 2.8 | 2.5 | 79.5 | 14.2 | 4.9 | 1.4 |
| Year 5 | 82.9 | 10.8 | 3.3 | 3.1 | 77.1 | 15.2 | 5.9 | 1.8 |
| Year 6 | 81.3 | 11.3 | 3.7 | 3.8 | 75.1 | 16.0 | 6.7 | 2.2 |
| Year 7 | 80.0 | 11.7 | 4.0 | 4.4 | 73.5 | 16.6 | 7.4 | 2.6 |
| Year 8 | 78.9 | 11.9 | 4.2 | 5.0 | 72.2 | 17.0 | 7.9 | 2.9 |
| Year 9 | 77.8 | 12.1 | 4.4 | 5.7 | 71.0 | 17.3 | 8.3 | 3.3 |
| Year 10 | 76.9 | 12.3 | 4.5 | 6.3 | 70.0 | 17.6 | 8.7 | 3.7 |

DS: depressive symptoms.

**Table S7** Estimated mean sojourn time in each DS state

| **DS state** | **Estimated mean sojourn time (years)** | **Standard errors** | **95% CI** | |
| --- | --- | --- | --- | --- |
|  |  |  | **Lower** | **Upper** |
| **Overall** |  |  |  |  |
| Non-DS | 5.744 | 0.090 | 5.570 | 5.923 |
| Mild-DS | 0.993 | 0.014 | 0.966 | 1.021 |
| Severe-DS | 1.390 | 0.033 | 1.327 | 1.457 |
| **Males** |  |  |  |  |
| Non-DS | 6.647 | 0.153 | 6.354 | 6.953 |
| Mild-DS | 0.959 | 0.021 | 0.919 | 1.000 |
| Severe-DS | 1.246 | 0.048 | 1.155 | 1.344 |
| **Females** |  |  |  |  |
| Non-DS | 4.947 | 0.104 | 4.747 | 5.155 |
| Mild-DS | 1.022 | 0.018 | 0.987 | 1.059 |
| Severe-DS | 1.540 | 0.044 | 1.456 | 1.629 |

DS: depressive symptoms.

**Table S8** Hazard ratios of covariates associated with transitions between DS states (age modeled as a continuous variable)

| **Factors** | **Deteriorate transition** | | | **Recovery transition** | | **Death transition** | | |
| --- | --- | --- | --- | --- | --- | --- | --- | --- |
|  | Non-DS→  Mild-DS | | Mild-DS→  Severe-DS | Mild-DS→  Non-DS | Severe-DS→  Mild-DS | Non-DS→  Death | Mild-DS→  Death | Severe-DS→  Death |
| **Sex** |  | |  |  |  |  |  |  |
| Males | 1 (ref) | | 1 (ref) | 1 (ref) | 1 (ref) | 1 (ref) | 1 (ref) | 1 (ref) |
| Females | **1.290 (1.200,1.386)** | | 1.056 (0.933,1.196) | **0.856 (0.798,0.917)** | **0.854 (0.767,0.951)** | **0.493 (0.409,0.595)** | 0.738 (0.420,1.297) | **0.414 (0.291,0.589)** |
| **Age, years** | **0.991 (0.987,0.996)** | | 1.006 (0.999,1.013) | **1.004 (1.001,1.008)** | **1.014 (1.008,1.020)** | **1.111 (1.100,1.123)** | **1.058 (1.025,1.091)** | **1.129 (1.104,1.156)** |
| **Education levels** |  | |  |  |  |  |  |  |
| Elementary below | 1 (ref) | | 1(ref) | 1 (ref) | 1 (ref) | 1 (ref) | 1 (ref) | 1 (ref) |
| Elementary | **0.784 (0.718,0.856)** | | **0.767 (0.663,0.887)** | 0.954 (0.878,1.037) | 1.031 (0.910,1.169) | 0.912 (0.752,1.107) | 0.563 (0.239,1.327) | 0.975 (0.648,1.468) |
| Middle school and above | **0.627 (0.574,0.685)** | | **0.746 (0.641,0.868)** | 0.987 (0.908,1.073) | 1.137 (0.998,1.294) | **0.705 (0.565,0.881)** | 1.261 (0.671,2.372) | 0.610 (0.309,1.207) |
| **Marital status** |  | |  |  |  |  |  |  |
| Married | 1 (ref) | | 1 (ref) | 1 (ref) | 1 (ref) | 1 (ref) | 1 (ref) | 1 (ref) |
| Others | 1.113 (0.999,1.241) | | 0.967 (0.821,1.139) | 0.962 (0.874,1.059) | **0.858 (0.752,0.980)** | **1.635 (1.351,1.979)** | 0.928 (0.452,1.903) | **1.595 (1.111,2.292)** |
| **Residential regions** |  | |  |  |  |  |  |  |
| Urban | 1 (ref) | | 1 (ref) | 1 (ref) | 1 (ref) | 1 (ref) | 1 (ref) | 1 (ref) |
| Rural | **1.399 (1.301,1.504)** | | **1.167 (1.031,1.322)** | 0.997 (0.931,1.067) | 1.018 (0.914,1.134) | 1.121 (0.942,1.335) | 1.245 (0.671,2.310) | 1.021 (0.695,1.498) |
| **Chronic disease conditions** | | |  |  |  |  |  |  |
| 0 | 1 (ref) | | 1 (ref) | 1 (ref) | 1 (ref) | 1 (ref) | 1 (ref) | 1 (ref) |
| 1-2 | **1.290 (1.186,1.404)** | | 1.046 (0.891,1.228) | 0.942 (0.866,1.023) | 0.897 (0.774,1.039) | **1.559 (1.247,1.949)** | 1.754 (0.820,3.749) | 1.527 (0.635,3.673) |
| ≥3 | **1.698 (1.543,1.870)** | | 0.963 (0.814,1.139) | **0.819 (0.748,0.898)** | **0.706 (0.608,0.820)** | **2.715 (2.152,3.427)** | 1.988 (0.919,4.300) | **3.073 (1.305,7.235)** |
| **Social participation** | |  |  |  |  |  |  |  |
| No | 1 (ref) | | 1 (ref) | 1 (ref) | 1 (ref) | 1 (ref) | 1 (ref) | 1 (ref) |
| Non-regular | 0.919 (0.843,1.002) | | 0.934 (0.810,1.077) | **0.906 (0.835,0.983)** | 0.998 (0.882,1.130) | 0.814 (0.659,1.005) | 0.750 (0.182,3.086) | **0.557 (0.328,0.946)** |
| Frequent | **0.851 (0.786,0.921)** | | 1.010 (0.878,1.161) | 0.930 (0.862,1.004) | **1.170 (1.035,1.323)** | **0.570 (0.456,0.713)** | **3.579 (1.573,8.143)** | 0.201 (0.036,1.126) |
| **Weight status** |  | |  |  |  |  |  |  |
| Normal | 1(ref) | | 1(ref) | 1(ref) | 1(ref) | 1(ref) | 1(ref) | 1(ref) |
| Underweight | **1.350 (1.139,1.600)** | | 1.052 (0.818,1.354) | 1.034 (0.880,1.215) | 0.984 (0.792,1.223) | **1.500 (1.132,1.988)** | **2.162 (1.069,4.372)** | 1.103 (0.584,2.084) |
| Overweight | 0.928 (0.860,1.001) | | 1.028 (0.901,1.173) | 0.984 (0.915,1.059) | **1.146 (1.022,1.286)** | 1.086 (0.908,1.298) | **0.353 (0.143,0.872)** | **1.531 (1.042,2.251)** |
| Obesity | 0.901 (0.809,1.004) | | 1.013 (0.847,1.212) | 0.974 (0.877,1.082) | 1.087 (0.928,1.273) | 0.870 (0.631,1.200) | 0.754 (0.277,2.056) | 1.301 (0.748,2.263) |

Values were hazard ratios (95% CI).

Boldfaced data indicate statistical signiﬁcance (P < 0.05).

DS: depressive symptoms.

**Table S9** Estimated probabilities of staying in non-DS over 10 years, stratified by sex and other factors

|  | | | **Non-DS → Non-DS** | | | | | | | | | |
| --- | --- | --- | --- | --- | --- | --- | --- | --- | --- | --- | --- | --- |
|  |  |  | Year 1 | Year 2 | Year 3 | Year 4 | Year 5 | Year 6 | Year 7 | Year 8 | Year 9 | Year 10 |
| **Age, years** | Males | 45-54 | 0.902 | 0.854 | 0.826 | 0.809 | 0.797 | 0.790 | 0.785 | 0.780 | 0.777 | 0.774 |
|  |  | 55-64 | 0.909 | 0.864 | 0.837 | 0.820 | 0.808 | 0.798 | 0.791 | 0.785 | 0.779 | 0.774 |
|  |  | ≥65 | 0.902 | 0.847 | 0.811 | 0.784 | 0.762 | 0.743 | 0.726 | 0.710 | 0.695 | 0.681 |
|  | Females | 45-54 | 0.872 | 0.806 | 0.766 | 0.741 | 0.725 | 0.714 | 0.707 | 0.702 | 0.699 | 0.696 |
|  |  | 55-64 | 0.883 | 0.824 | 0.788 | 0.765 | 0.750 | 0.740 | 0.732 | 0.727 | 0.722 | 0.719 |
|  |  | ≥65 | 0.886 | 0.824 | 0.786 | 0.761 | 0.742 | 0.728 | 0.717 | 0.708 | 0.700 | 0.692 |
| **Chronic disease conditions** | Males | 0 | 0.902 | 0.854 | 0.826 | 0.809 | 0.797 | 0.790 | 0.785 | 0.780 | 0.777 | 0.774 |
|  |  | 1-2 | 0.874 | 0.810 | 0.773 | 0.749 | 0.734 | 0.723 | 0.715 | 0.709 | 0.704 | 0.700 |
|  |  | ≥3 | 0.831 | 0.745 | 0.694 | 0.661 | 0.638 | 0.622 | 0.610 | 0.601 | 0.593 | 0.587 |
|  | Females | 0 | 0.872 | 0.806 | 0.766 | 0.741 | 0.725 | 0.714 | 0.707 | 0.702 | 0.699 | 0.696 |
|  |  | 1-2 | 0.836 | 0.752 | 0.702 | 0.670 | 0.649 | 0.635 | 0.626 | 0.619 | 0.615 | 0.611 |
|  |  | ≥3 | 0.785 | 0.676 | 0.612 | 0.571 | 0.544 | 0.526 | 0.513 | 0.505 | 0.498 | 0.493 |
| **Social participation** | Males | No | 0.902 | 0.854 | 0.826 | 0.809 | 0.797 | 0.790 | 0.785 | 0.780 | 0.777 | 0.774 |
|  |  | Non-regular | 0.908 | 0.860 | 0.831 | 0.814 | 0.802 | 0.795 | 0.790 | 0.786 | 0.783 | 0.780 |
|  |  | Frequent | 0.915 | 0.871 | 0.845 | 0.830 | 0.819 | 0.813 | 0.808 | 0.804 | 0.801 | 0.799 |
|  | Females | No | 0.872 | 0.806 | 0.766 | 0.741 | 0.725 | 0.714 | 0.707 | 0.702 | 0.699 | 0.696 |
|  |  | Non-regular | 0.879 | 0.813 | 0.773 | 0.747 | 0.730 | 0.719 | 0.711 | 0.706 | 0.703 | 0.700 |
|  |  | Frequent | 0.888 | 0.827 | 0.790 | 0.766 | 0.751 | 0.741 | 0.734 | 0.729 | 0.726 | 0.723 |
| **Weight status** | Males | Normal | 0.902 | 0.854 | 0.826 | 0.809 | 0.797 | 0.790 | 0.785 | 0.780 | 0.777 | 0.774 |
|  |  | Underweight | 0.872 | 0.812 | 0.778 | 0.757 | 0.743 | 0.734 | 0.727 | 0.722 | 0.717 | 0.713 |
|  |  | Overweight | 0.908 | 0.862 | 0.836 | 0.819 | 0.809 | 0.802 | 0.797 | 0.794 | 0.790 | 0.788 |
|  |  | Obesity | 0.911 | 0.866 | 0.840 | 0.823 | 0.813 | 0.806 | 0.801 | 0.798 | 0.795 | 0.792 |
|  | Females | Normal | 0.872 | 0.806 | 0.766 | 0.741 | 0.725 | 0.714 | 0.707 | 0.702 | 0.699 | 0.696 |
|  |  | Underweight | 0.834 | 0.753 | 0.707 | 0.679 | 0.661 | 0.649 | 0.642 | 0.636 | 0.632 | 0.629 |
|  |  | Overweight | 0.880 | 0.816 | 0.779 | 0.755 | 0.741 | 0.731 | 0.725 | 0.720 | 0.717 | 0.715 |
|  |  | Obesity | 0.883 | 0.821 | 0.783 | 0.759 | 0.744 | 0.734 | 0.727 | 0.723 | 0.720 | 0.717 |

Model adjusted for age, education levels, residential regions, marital status, chronic disease conditions, social participation, and weight status.

DS: depressive symptoms.

**Table S10** Estimated transition probabilities from non-DS to mild-DS over 10 years, stratified by sex and other factors

|  | | | **Non-DS → Mild-DS** | | | | | | | | | |
| --- | --- | --- | --- | --- | --- | --- | --- | --- | --- | --- | --- | --- |
|  |  |  | Year 1 | Year 2 | Year 3 | Year 4 | Year 5 | Year 6 | Year 7 | Year 8 | Year 9 | Year 10 |
| **Age, years** | Males | 45-54 | 0.081 | 0.109 | 0.121 | 0.128 | 0.132 | 0.134 | 0.135 | 0.136 | 0.136 | 0.136 |
|  |  | 55-64 | 0.073 | 0.096 | 0.106 | 0.112 | 0.114 | 0.115 | 0.116 | 0.116 | 0.116 | 0.115 |
|  |  | ≥65 | 0.065 | 0.086 | 0.094 | 0.098 | 0.098 | 0.098 | 0.097 | 0.095 | 0.094 | 0.092 |
|  | Females | 45-54 | 0.106 | 0.143 | 0.161 | 0.170 | 0.176 | 0.179 | 0.181 | 0.183 | 0.183 | 0.184 |
|  |  | 55-64 | 0.096 | 0.129 | 0.143 | 0.151 | 0.156 | 0.158 | 0.160 | 0.161 | 0.161 | 0.161 |
|  |  | ≥65 | 0.087 | 0.116 | 0.130 | 0.137 | 0.140 | 0.142 | 0.142 | 0.142 | 0.141 | 0.140 |
| **Chronic disease conditions** | Males | 0 | 0.081 | 0.109 | 0.121 | 0.128 | 0.132 | 0.134 | 0.135 | 0.136 | 0.136 | 0.136 |
|  |  | 1-2 | 0.103 | 0.137 | 0.152 | 0.159 | 0.164 | 0.166 | 0.167 | 0.167 | 0.167 | 0.167 |
|  |  | ≥3 | 0.137 | 0.180 | 0.198 | 0.206 | 0.210 | 0.211 | 0.212 | 0.212 | 0.211 | 0.210 |
|  | Females | 0 | 0.106 | 0.143 | 0.161 | 0.170 | 0.176 | 0.179 | 0.181 | 0.183 | 0.183 | 0.184 |
|  |  | 1-2 | 0.134 | 0.178 | 0.196 | 0.206 | 0.212 | 0.216 | 0.218 | 0.219 | 0.219 | 0.219 |
|  |  | ≥3 | 0.176 | 0.230 | 0.250 | 0.258 | 0.263 | 0.265 | 0.266 | 0.266 | 0.266 | 0.265 |
| **Social participation** | Males | No | 0.081 | 0.109 | 0.121 | 0.128 | 0.132 | 0.134 | 0.135 | 0.136 | 0.136 | 0.136 |
|  |  | Non-regular | 0.078 | 0.107 | 0.121 | 0.129 | 0.133 | 0.136 | 0.138 | 0.138 | 0.139 | 0.139 |
|  |  | Frequent | 0.071 | 0.098 | 0.111 | 0.119 | 0.123 | 0.125 | 0.126 | 0.127 | 0.127 | 0.127 |
|  | Females | No | 0.106 | 0.143 | 0.161 | 0.170 | 0.176 | 0.179 | 0.181 | 0.183 | 0.183 | 0.184 |
|  |  | Non-regular | 0.102 | 0.141 | 0.160 | 0.171 | 0.177 | 0.181 | 0.184 | 0.186 | 0.187 | 0.187 |
|  |  | Frequent | 0.093 | 0.130 | 0.148 | 0.159 | 0.165 | 0.169 | 0.171 | 0.172 | 0.173 | 0.174 |
| **Weight status** | Males | Normal | 0.081 | 0.109 | 0.121 | 0.128 | 0.132 | 0.134 | 0.135 | 0.136 | 0.136 | 0.136 |
|  |  | Underweight | 0.104 | 0.136 | 0.149 | 0.156 | 0.159 | 0.161 | 0.162 | 0.162 | 0.161 | 0.161 |
|  |  | Overweight | 0.076 | 0.103 | 0.116 | 0.123 | 0.127 | 0.129 | 0.130 | 0.130 | 0.131 | 0.130 |
|  |  | Obesity | 0.074 | 0.101 | 0.114 | 0.120 | 0.124 | 0.126 | 0.128 | 0.128 | 0.128 | 0.128 |
|  | Females | Normal | 0.106 | 0.143 | 0.161 | 0.170 | 0.176 | 0.179 | 0.181 | 0.183 | 0.183 | 0.184 |
|  |  | Underweight | 0.135 | 0.177 | 0.195 | 0.204 | 0.209 | 0.212 | 0.213 | 0.214 | 0.214 | 0.214 |
|  |  | Overweight | 0.100 | 0.137 | 0.154 | 0.164 | 0.170 | 0.174 | 0.176 | 0.177 | 0.178 | 0.179 |
|  |  | Obesity | 0.098 | 0.134 | 0.151 | 0.161 | 0.167 | 0.170 | 0.173 | 0.174 | 0.175 | 0.175 |

Model adjusted for age, education levels, residential regions, marital status, chronic disease conditions, social participation, and weight status.

DS: depressive symptoms.

**Table S11** Estimated transition probabilities from non-DS to severe-DS over 10 years, stratified by sex and other factors

|  | | | **Non-DS → Severe-DS** | | | | | | | | | |
| --- | --- | --- | --- | --- | --- | --- | --- | --- | --- | --- | --- | --- |
|  |  |  | Year 1 | Year 2 | Year 3 | Year 4 | Year 5 | Year 6 | Year 7 | Year 8 | Year 9 | Year 10 |
| **Age, years** | Males | 45-54 | 0.014 | 0.032 | 0.045 | 0.053 | 0.058 | 0.060 | 0.062 | 0.063 | 0.063 | 0.064 |
|  |  | 55-64 | 0.012 | 0.028 | 0.038 | 0.045 | 0.049 | 0.051 | 0.052 | 0.053 | 0.053 | 0.053 |
|  |  | ≥65 | 0.012 | 0.026 | 0.034 | 0.038 | 0.040 | 0.041 | 0.041 | 0.041 | 0.040 | 0.039 |
|  | Females | 45-54 | 0.020 | 0.048 | 0.070 | 0.084 | 0.093 | 0.099 | 0.103 | 0.105 | 0.106 | 0.107 |
|  |  | 55-64 | 0.018 | 0.042 | 0.060 | 0.072 | 0.080 | 0.085 | 0.088 | 0.090 | 0.091 | 0.092 |
|  |  | ≥65 | 0.018 | 0.040 | 0.055 | 0.064 | 0.070 | 0.073 | 0.075 | 0.075 | 0.075 | 0.075 |
| **Chronic disease conditions** | Males | 0 | 0.014 | 0.032 | 0.045 | 0.053 | 0.058 | 0.060 | 0.062 | 0.063 | 0.063 | 0.064 |
|  |  | 1-2 | 0.020 | 0.045 | 0.063 | 0.074 | 0.081 | 0.085 | 0.088 | 0.089 | 0.090 | 0.090 |
|  |  | ≥3 | 0.025 | 0.060 | 0.087 | 0.104 | 0.115 | 0.122 | 0.126 | 0.128 | 0.129 | 0.129 |
|  | Females | 0 | 0.020 | 0.048 | 0.070 | 0.084 | 0.093 | 0.099 | 0.103 | 0.105 | 0.106 | 0.107 |
|  |  | 1-2 | 0.028 | 0.067 | 0.096 | 0.115 | 0.128 | 0.136 | 0.141 | 0.144 | 0.146 | 0.147 |
|  |  | ≥3 | 0.035 | 0.087 | 0.128 | 0.156 | 0.175 | 0.188 | 0.195 | 0.200 | 0.203 | 0.205 |
| **Social participation** | Males | No | 0.014 | 0.032 | 0.045 | 0.053 | 0.058 | 0.060 | 0.062 | 0.063 | 0.063 | 0.064 |
|  |  | Non-regular | 0.013 | 0.029 | 0.042 | 0.050 | 0.054 | 0.057 | 0.059 | 0.060 | 0.061 | 0.061 |
|  |  | Frequent | 0.012 | 0.027 | 0.037 | 0.043 | 0.047 | 0.049 | 0.051 | 0.051 | 0.052 | 0.052 |
|  | Females | No | 0.020 | 0.048 | 0.070 | 0.084 | 0.093 | 0.099 | 0.103 | 0.105 | 0.106 | 0.107 |
|  |  | Non-regular | 0.018 | 0.044 | 0.064 | 0.078 | 0.087 | 0.094 | 0.098 | 0.100 | 0.102 | 0.103 |
|  |  | Frequent | 0.017 | 0.041 | 0.058 | 0.069 | 0.077 | 0.082 | 0.084 | 0.086 | 0.087 | 0.088 |
| **Weight status** | Males | Normal | 0.014 | 0.032 | 0.045 | 0.053 | 0.058 | 0.060 | 0.062 | 0.063 | 0.063 | 0.064 |
|  |  | Underweight | 0.020 | 0.043 | 0.059 | 0.069 | 0.074 | 0.077 | 0.079 | 0.080 | 0.080 | 0.080 |
|  |  | Overweight | 0.013 | 0.029 | 0.040 | 0.047 | 0.051 | 0.053 | 0.054 | 0.055 | 0.055 | 0.055 |
|  |  | Obesity | 0.013 | 0.029 | 0.040 | 0.047 | 0.052 | 0.054 | 0.055 | 0.056 | 0.057 | 0.057 |
|  | Females | Normal | 0.020 | 0.048 | 0.070 | 0.084 | 0.093 | 0.099 | 0.103 | 0.105 | 0.106 | 0.107 |
|  |  | Underweight | 0.028 | 0.065 | 0.091 | 0.108 | 0.118 | 0.125 | 0.128 | 0.131 | 0.132 | 0.132 |
|  |  | Overweight | 0.019 | 0.044 | 0.063 | 0.075 | 0.083 | 0.088 | 0.091 | 0.092 | 0.093 | 0.094 |
|  |  | Obesity | 0.019 | 0.044 | 0.063 | 0.076 | 0.084 | 0.089 | 0.092 | 0.095 | 0.096 | 0.097 |

Model adjusted for age, education levels, residential regions, marital status, chronic disease conditions, social participation, and weight status.

DS: depressive symptoms.

**Table S12** Estimated transition probabilities from non-DS to death over 10 years, stratified by sex and other factors

|  | | | **Non-DS → Death** | | | | | | | | | |
| --- | --- | --- | --- | --- | --- | --- | --- | --- | --- | --- | --- | --- |
|  |  |  | Year 1 | Year 2 | Year 3 | Year 4 | Year 5 | Year 6 | Year 7 | Year 8 | Year 9 | Year 10 |
| **Age, years** | Males | 45-54 | 0.003 | 0.005 | 0.008 | 0.010 | 0.013 | 0.016 | 0.018 | 0.021 | 0.024 | 0.026 |
|  |  | 55-64 | 0.006 | 0.012 | 0.018 | 0.024 | 0.029 | 0.035 | 0.041 | 0.047 | 0.052 | 0.058 |
|  |  | ≥65 | 0.021 | 0.041 | 0.061 | 0.080 | 0.099 | 0.118 | 0.136 | 0.154 | 0.171 | 0.188 |
|  | Females | 45-54 | 0.001 | 0.003 | 0.004 | 0.005 | 0.006 | 0.008 | 0.009 | 0.010 | 0.012 | 0.013 |
|  |  | 55-64 | 0.003 | 0.006 | 0.009 | 0.011 | 0.014 | 0.017 | 0.020 | 0.023 | 0.025 | 0.028 |
|  |  | ≥65 | 0.010 | 0.019 | 0.029 | 0.038 | 0.048 | 0.057 | 0.066 | 0.075 | 0.084 | 0.093 |
| **Chronic disease conditions** | Males | 0 | 0.003 | 0.005 | 0.008 | 0.010 | 0.013 | 0.016 | 0.018 | 0.021 | 0.024 | 0.026 |
|  |  | 1-2 | 0.004 | 0.008 | 0.013 | 0.017 | 0.021 | 0.026 | 0.030 | 0.034 | 0.039 | 0.043 |
|  |  | ≥3 | 0.007 | 0.015 | 0.022 | 0.029 | 0.037 | 0.044 | 0.052 | 0.059 | 0.067 | 0.074 |
|  | Females | 0 | 0.001 | 0.003 | 0.004 | 0.005 | 0.006 | 0.008 | 0.009 | 0.010 | 0.012 | 0.013 |
|  |  | 1-2 | 0.002 | 0.004 | 0.006 | 0.008 | 0.011 | 0.013 | 0.015 | 0.017 | 0.020 | 0.022 |
|  |  | ≥3 | 0.003 | 0.007 | 0.010 | 0.014 | 0.018 | 0.022 | 0.025 | 0.029 | 0.033 | 0.037 |
| **Social participation** | Males | No | 0.003 | 0.005 | 0.008 | 0.010 | 0.013 | 0.016 | 0.018 | 0.021 | 0.024 | 0.026 |
|  |  | Non-regular | 0.002 | 0.004 | 0.006 | 0.008 | 0.010 | 0.012 | 0.014 | 0.016 | 0.018 | 0.019 |
|  |  | Frequent | 0.002 | 0.004 | 0.006 | 0.008 | 0.011 | 0.013 | 0.015 | 0.018 | 0.020 | 0.022 |
|  | Females | No | 0.001 | 0.003 | 0.004 | 0.005 | 0.006 | 0.008 | 0.009 | 0.010 | 0.012 | 0.013 |
|  |  | Non-regular | 0.001 | 0.002 | 0.003 | 0.004 | 0.005 | 0.006 | 0.007 | 0.008 | 0.009 | 0.010 |
|  |  | Frequent | 0.001 | 0.002 | 0.004 | 0.005 | 0.007 | 0.009 | 0.010 | 0.012 | 0.014 | 0.015 |
| **Weight status** | Males | Normal | 0.003 | 0.005 | 0.008 | 0.010 | 0.013 | 0.016 | 0.018 | 0.021 | 0.024 | 0.026 |
|  |  | Underweight | 0.005 | 0.009 | 0.014 | 0.018 | 0.023 | 0.028 | 0.032 | 0.037 | 0.041 | 0.046 |
|  |  | Overweight | 0.003 | 0.005 | 0.008 | 0.011 | 0.013 | 0.016 | 0.019 | 0.021 | 0.024 | 0.027 |
|  |  | Obesity | 0.002 | 0.004 | 0.007 | 0.009 | 0.011 | 0.013 | 0.016 | 0.018 | 0.020 | 0.022 |
|  | Females | Normal | 0.001 | 0.003 | 0.004 | 0.005 | 0.006 | 0.008 | 0.009 | 0.010 | 0.012 | 0.013 |
|  |  | Underweight | 0.002 | 0.005 | 0.007 | 0.009 | 0.012 | 0.014 | 0.017 | 0.019 | 0.021 | 0.024 |
|  |  | Overweight | 0.001 | 0.002 | 0.004 | 0.005 | 0.006 | 0.007 | 0.009 | 0.010 | 0.011 | 0.012 |
|  |  | Obesity | 0.001 | 0.002 | 0.003 | 0.004 | 0.005 | 0.006 | 0.008 | 0.009 | 0.010 | 0.011 |

Model adjusted for age, education levels, residential regions, marital status, chronic disease conditions, social participation, and weight status.

DS: depressive symptoms.

**Table S13** Estimated transition probabilities from mild-DS to non-DS over 10 years, stratified by sex and other factors

|  | | | **Mild-DS → Non-DS** | | | | | | | | | |
| --- | --- | --- | --- | --- | --- | --- | --- | --- | --- | --- | --- | --- |
|  |  |  | Year 1 | Year 2 | Year 3 | Year 4 | Year 5 | Year 6 | Year 7 | Year 8 | Year 9 | Year 10 |
| **Age, years** | Males | 45-54 | 0.459 | 0.617 | 0.689 | 0.727 | 0.748 | 0.760 | 0.766 | 0.769 | 0.770 | 0.770 |
|  |  | 55-64 | 0.486 | 0.645 | 0.714 | 0.748 | 0.765 | 0.774 | 0.777 | 0.777 | 0.775 | 0.772 |
|  |  | ≥65 | 0.485 | 0.639 | 0.701 | 0.726 | 0.733 | 0.730 | 0.722 | 0.711 | 0.698 | 0.685 |
|  | Females | 45-54 | 0.398 | 0.538 | 0.602 | 0.637 | 0.659 | 0.672 | 0.679 | 0.684 | 0.687 | 0.688 |
|  |  | 55-64 | 0.425 | 0.570 | 0.634 | 0.668 | 0.688 | 0.700 | 0.707 | 0.711 | 0.712 | 0.712 |
|  |  | ≥65 | 0.426 | 0.572 | 0.638 | 0.672 | 0.689 | 0.697 | 0.699 | 0.698 | 0.694 | 0.689 |
| **Chronic disease conditions** | Males | 0 | 0.459 | 0.617 | 0.689 | 0.727 | 0.748 | 0.760 | 0.766 | 0.769 | 0.770 | 0.770 |
|  |  | 1-2 | 0.427 | 0.569 | 0.631 | 0.663 | 0.681 | 0.690 | 0.695 | 0.696 | 0.696 | 0.695 |
|  |  | ≥3 | 0.378 | 0.499 | 0.547 | 0.569 | 0.580 | 0.585 | 0.586 | 0.585 | 0.583 | 0.580 |
|  | Females | 0 | 0.398 | 0.538 | 0.602 | 0.637 | 0.659 | 0.672 | 0.679 | 0.684 | 0.687 | 0.688 |
|  |  | 1-2 | 0.367 | 0.488 | 0.540 | 0.567 | 0.583 | 0.593 | 0.598 | 0.601 | 0.603 | 0.603 |
|  |  | ≥3 | 0.322 | 0.420 | 0.456 | 0.472 | 0.480 | 0.484 | 0.485 | 0.486 | 0.486 | 0.485 |
| **Social participation** | Males | No | 0.459 | 0.617 | 0.689 | 0.727 | 0.748 | 0.760 | 0.766 | 0.769 | 0.770 | 0.770 |
|  |  | Non-regular | 0.433 | 0.597 | 0.675 | 0.719 | 0.744 | 0.759 | 0.767 | 0.772 | 0.774 | 0.775 |
|  |  | Frequent | 0.441 | 0.609 | 0.691 | 0.736 | 0.761 | 0.776 | 0.783 | 0.787 | 0.789 | 0.789 |
|  | Females | No | 0.398 | 0.538 | 0.602 | 0.637 | 0.659 | 0.672 | 0.679 | 0.684 | 0.687 | 0.688 |
|  |  | Non-regular | 0.375 | 0.519 | 0.589 | 0.629 | 0.653 | 0.668 | 0.678 | 0.684 | 0.688 | 0.690 |
|  |  | Frequent | 0.383 | 0.532 | 0.606 | 0.649 | 0.675 | 0.691 | 0.701 | 0.706 | 0.709 | 0.711 |
| **Weight status** | Males | Normal | 0.459 | 0.617 | 0.689 | 0.727 | 0.748 | 0.760 | 0.766 | 0.769 | 0.770 | 0.770 |
|  |  | Underweight | 0.457 | 0.599 | 0.659 | 0.688 | 0.704 | 0.711 | 0.714 | 0.714 | 0.713 | 0.710 |
|  |  | Overweight | 0.457 | 0.621 | 0.699 | 0.740 | 0.763 | 0.775 | 0.782 | 0.785 | 0.785 | 0.785 |
|  |  | Obesity | 0.455 | 0.619 | 0.696 | 0.738 | 0.762 | 0.775 | 0.782 | 0.786 | 0.787 | 0.788 |
|  | Females | Normal | 0.398 | 0.538 | 0.602 | 0.637 | 0.659 | 0.672 | 0.679 | 0.684 | 0.687 | 0.688 |
|  |  | Underweight | 0.394 | 0.517 | 0.568 | 0.594 | 0.609 | 0.617 | 0.621 | 0.624 | 0.624 | 0.624 |
|  |  | Overweight | 0.396 | 0.542 | 0.613 | 0.653 | 0.677 | 0.691 | 0.700 | 0.705 | 0.707 | 0.709 |
|  |  | Obesity | 0.395 | 0.540 | 0.611 | 0.650 | 0.675 | 0.689 | 0.699 | 0.704 | 0.707 | 0.709 |

Model adjusted for age, education levels, residential regions, marital status, chronic disease conditions, social participation, and weight status.

DS: depressive symptoms.

**Table S14** Estimated probabilities of staying in mild-DS over 10 years, stratified by sex and other factors

|  | | | **Mild-DS → Mild-DS** | | | | | | | | | |
| --- | --- | --- | --- | --- | --- | --- | --- | --- | --- | --- | --- | --- |
|  |  |  | Year 1 | Year 2 | Year 3 | Year 4 | Year 5 | Year 6 | Year 7 | Year 8 | Year 9 | Year 10 |
| **Age, years** | Males | 45-54 | 0.386 | 0.235 | 0.187 | 0.165 | 0.154 | 0.147 | 0.143 | 0.140 | 0.138 | 0.137 |
|  |  | 55-64 | 0.366 | 0.213 | 0.165 | 0.145 | 0.134 | 0.127 | 0.123 | 0.120 | 0.118 | 0.117 |
|  |  | ≥65 | 0.354 | 0.206 | 0.156 | 0.131 | 0.117 | 0.109 | 0.103 | 0.099 | 0.096 | 0.094 |
|  | Females | 45-54 | 0.420 | 0.274 | 0.229 | 0.210 | 0.201 | 0.195 | 0.191 | 0.189 | 0.187 | 0.186 |
|  |  | 55-64 | 0.403 | 0.253 | 0.207 | 0.188 | 0.179 | 0.173 | 0.169 | 0.167 | 0.165 | 0.163 |
|  |  | ≥65 | 0.389 | 0.246 | 0.199 | 0.177 | 0.165 | 0.157 | 0.152 | 0.148 | 0.145 | 0.143 |
| **Chronic disease conditions** | Males | 0 | 0.386 | 0.235 | 0.187 | 0.165 | 0.154 | 0.147 | 0.143 | 0.140 | 0.138 | 0.137 |
|  |  | 1-2 | 0.399 | 0.256 | 0.212 | 0.194 | 0.184 | 0.178 | 0.174 | 0.172 | 0.170 | 0.168 |
|  |  | ≥3 | 0.438 | 0.294 | 0.251 | 0.235 | 0.227 | 0.222 | 0.219 | 0.216 | 0.213 | 0.211 |
|  | Females | 0 | 0.420 | 0.274 | 0.229 | 0.210 | 0.201 | 0.195 | 0.191 | 0.189 | 0.187 | 0.186 |
|  |  | 1-2 | 0.431 | 0.293 | 0.254 | 0.240 | 0.233 | 0.229 | 0.226 | 0.224 | 0.222 | 0.221 |
|  |  | ≥3 | 0.470 | 0.331 | 0.292 | 0.280 | 0.275 | 0.273 | 0.271 | 0.269 | 0.268 | 0.267 |
| **Social participation** | Males | No | 0.386 | 0.235 | 0.187 | 0.165 | 0.154 | 0.147 | 0.143 | 0.140 | 0.138 | 0.137 |
|  |  | Non-regular | 0.415 | 0.256 | 0.201 | 0.176 | 0.162 | 0.153 | 0.148 | 0.145 | 0.143 | 0.141 |
|  |  | Frequent | 0.405 | 0.249 | 0.192 | 0.165 | 0.149 | 0.141 | 0.135 | 0.132 | 0.130 | 0.128 |
|  | Females | No | 0.420 | 0.274 | 0.229 | 0.210 | 0.201 | 0.195 | 0.191 | 0.189 | 0.187 | 0.186 |
|  |  | Non-regular | 0.448 | 0.294 | 0.243 | 0.221 | 0.209 | 0.202 | 0.197 | 0.194 | 0.192 | 0.191 |
|  |  | Frequent | 0.438 | 0.287 | 0.235 | 0.211 | 0.197 | 0.189 | 0.183 | 0.180 | 0.177 | 0.176 |
| **Weight status** | Males | Normal | 0.386 | 0.235 | 0.187 | 0.165 | 0.154 | 0.147 | 0.143 | 0.140 | 0.138 | 0.137 |
|  |  | Underweight | 0.379 | 0.242 | 0.202 | 0.185 | 0.176 | 0.171 | 0.167 | 0.165 | 0.163 | 0.162 |
|  |  | Overweight | 0.392 | 0.241 | 0.188 | 0.164 | 0.150 | 0.143 | 0.138 | 0.135 | 0.133 | 0.132 |
|  |  | Obesity | 0.392 | 0.239 | 0.186 | 0.162 | 0.149 | 0.141 | 0.136 | 0.133 | 0.131 | 0.130 |
|  | Females | Normal | 0.420 | 0.274 | 0.229 | 0.210 | 0.201 | 0.195 | 0.191 | 0.189 | 0.187 | 0.186 |
|  |  | Underweight | 0.414 | 0.282 | 0.246 | 0.233 | 0.226 | 0.222 | 0.220 | 0.218 | 0.216 | 0.215 |
|  |  | Overweight | 0.425 | 0.280 | 0.232 | 0.210 | 0.199 | 0.192 | 0.187 | 0.184 | 0.182 | 0.181 |
|  |  | Obesity | 0.425 | 0.277 | 0.228 | 0.207 | 0.196 | 0.189 | 0.184 | 0.181 | 0.179 | 0.178 |

Model adjusted for age, education levels, residential regions, marital status, chronic disease conditions, social participation, and weight status.

DS: depressive symptoms.

**Table S15** Estimated transition probabilities from mild-DS to severe-DS over 10 years, stratified by sex and other factors

|  | | | **Mild-DS → Severe-DS** | | | | | | | | | |
| --- | --- | --- | --- | --- | --- | --- | --- | --- | --- | --- | --- | --- |
|  |  |  | Year 1 | Year 2 | Year 3 | Year 4 | Year 5 | Year 6 | Year 7 | Year 8 | Year 9 | Year 10 |
| **Age, years** | Males | 45-54 | 0.153 | 0.143 | 0.116 | 0.097 | 0.084 | 0.077 | 0.072 | 0.069 | 0.067 | 0.066 |
|  |  | 55-64 | 0.143 | 0.131 | 0.105 | 0.086 | 0.073 | 0.066 | 0.061 | 0.058 | 0.056 | 0.055 |
|  |  | ≥65 | 0.146 | 0.121 | 0.090 | 0.070 | 0.058 | 0.051 | 0.047 | 0.044 | 0.042 | 0.041 |
|  | Females | 45-54 | 0.180 | 0.186 | 0.165 | 0.147 | 0.134 | 0.125 | 0.119 | 0.116 | 0.113 | 0.112 |
|  |  | 55-64 | 0.169 | 0.172 | 0.151 | 0.132 | 0.119 | 0.110 | 0.104 | 0.100 | 0.098 | 0.096 |
|  |  | ≥65 | 0.177 | 0.165 | 0.136 | 0.115 | 0.101 | 0.092 | 0.086 | 0.082 | 0.080 | 0.078 |
| **Chronic disease conditions** | Males | 0 | 0.153 | 0.143 | 0.116 | 0.097 | 0.084 | 0.077 | 0.072 | 0.069 | 0.067 | 0.066 |
|  |  | 1-2 | 0.170 | 0.167 | 0.143 | 0.125 | 0.112 | 0.104 | 0.099 | 0.096 | 0.094 | 0.092 |
|  |  | ≥3 | 0.178 | 0.193 | 0.180 | 0.165 | 0.155 | 0.147 | 0.142 | 0.138 | 0.135 | 0.133 |
|  | Females | 0 | 0.180 | 0.186 | 0.165 | 0.147 | 0.134 | 0.125 | 0.119 | 0.116 | 0.113 | 0.112 |
|  |  | 1-2 | 0.199 | 0.214 | 0.199 | 0.183 | 0.172 | 0.164 | 0.159 | 0.156 | 0.154 | 0.152 |
|  |  | ≥3 | 0.205 | 0.242 | 0.241 | 0.233 | 0.226 | 0.221 | 0.217 | 0.215 | 0.213 | 0.211 |
| **Social participation** | Males | No | 0.153 | 0.143 | 0.116 | 0.097 | 0.084 | 0.077 | 0.072 | 0.069 | 0.067 | 0.066 |
|  |  | Non-regular | 0.150 | 0.143 | 0.118 | 0.098 | 0.085 | 0.076 | 0.071 | 0.067 | 0.065 | 0.064 |
|  |  | Frequent | 0.148 | 0.132 | 0.104 | 0.084 | 0.071 | 0.064 | 0.059 | 0.056 | 0.054 | 0.053 |
|  | Females | No | 0.180 | 0.186 | 0.165 | 0.147 | 0.134 | 0.125 | 0.119 | 0.116 | 0.113 | 0.112 |
|  |  | Non-regular | 0.175 | 0.184 | 0.165 | 0.146 | 0.132 | 0.123 | 0.117 | 0.113 | 0.110 | 0.108 |
|  |  | Frequent | 0.175 | 0.174 | 0.149 | 0.129 | 0.115 | 0.105 | 0.099 | 0.095 | 0.093 | 0.091 |
| **Weight status** | Males | Normal | 0.153 | 0.143 | 0.116 | 0.097 | 0.084 | 0.077 | 0.072 | 0.069 | 0.067 | 0.066 |
|  |  | Underweight | 0.160 | 0.150 | 0.125 | 0.108 | 0.097 | 0.091 | 0.087 | 0.084 | 0.082 | 0.081 |
|  |  | Overweight | 0.150 | 0.133 | 0.105 | 0.086 | 0.074 | 0.066 | 0.062 | 0.059 | 0.058 | 0.057 |
|  |  | Obesity | 0.151 | 0.138 | 0.110 | 0.090 | 0.077 | 0.069 | 0.065 | 0.062 | 0.060 | 0.059 |
|  | Females | Normal | 0.180 | 0.186 | 0.165 | 0.147 | 0.134 | 0.125 | 0.119 | 0.116 | 0.113 | 0.112 |
|  |  | Underweight | 0.189 | 0.196 | 0.178 | 0.162 | 0.152 | 0.145 | 0.141 | 0.138 | 0.136 | 0.135 |
|  |  | Overweight | 0.178 | 0.176 | 0.152 | 0.132 | 0.119 | 0.110 | 0.105 | 0.101 | 0.099 | 0.098 |
|  |  | Obesity | 0.179 | 0.180 | 0.157 | 0.138 | 0.124 | 0.115 | 0.109 | 0.105 | 0.102 | 0.101 |

Model adjusted for age, education levels, residential regions, marital status, chronic disease conditions, social participation, and weight status.

DS: depressive symptoms.

**Table S16** Estimated transition probabilities from mild-DS to death over 10 years, stratified by sex and other factors

|  | | | **Mild-DS → Death** | | | | | | | | | |
| --- | --- | --- | --- | --- | --- | --- | --- | --- | --- | --- | --- | --- |
|  |  |  | Year 1 | Year 2 | Year 3 | Year 4 | Year 5 | Year 6 | Year 7 | Year 8 | Year 9 | Year 10 |
| **Age, years** | Males | 45-54 | 0.003 | 0.005 | 0.008 | 0.011 | 0.014 | 0.016 | 0.019 | 0.022 | 0.024 | 0.027 |
|  |  | 55-64 | 0.005 | 0.010 | 0.016 | 0.022 | 0.028 | 0.034 | 0.039 | 0.045 | 0.051 | 0.056 |
|  |  | ≥65 | 0.015 | 0.034 | 0.053 | 0.073 | 0.091 | 0.110 | 0.128 | 0.146 | 0.164 | 0.181 |
|  | Females | 45-54 | 0.002 | 0.003 | 0.004 | 0.006 | 0.007 | 0.009 | 0.010 | 0.011 | 0.013 | 0.014 |
|  |  | 55-64 | 0.003 | 0.006 | 0.008 | 0.011 | 0.014 | 0.017 | 0.020 | 0.022 | 0.025 | 0.028 |
|  |  | ≥65 | 0.008 | 0.017 | 0.026 | 0.036 | 0.045 | 0.054 | 0.063 | 0.072 | 0.081 | 0.090 |
| **Chronic disease conditions** | Males | 0 | 0.003 | 0.005 | 0.008 | 0.011 | 0.014 | 0.016 | 0.019 | 0.022 | 0.024 | 0.027 |
|  |  | 1-2 | 0.004 | 0.009 | 0.013 | 0.018 | 0.023 | 0.027 | 0.031 | 0.036 | 0.040 | 0.044 |
|  |  | ≥3 | 0.006 | 0.014 | 0.022 | 0.030 | 0.038 | 0.046 | 0.053 | 0.061 | 0.069 | 0.076 |
|  | Females | 0 | 0.002 | 0.003 | 0.004 | 0.006 | 0.007 | 0.009 | 0.010 | 0.011 | 0.013 | 0.014 |
|  |  | 1-2 | 0.002 | 0.005 | 0.007 | 0.010 | 0.012 | 0.014 | 0.016 | 0.019 | 0.021 | 0.023 |
|  |  | ≥3 | 0.003 | 0.007 | 0.011 | 0.015 | 0.019 | 0.023 | 0.026 | 0.030 | 0.034 | 0.038 |
| **Social participation** | Males | No | 0.003 | 0.005 | 0.008 | 0.011 | 0.014 | 0.016 | 0.019 | 0.022 | 0.024 | 0.027 |
|  |  | Non-regular | 0.002 | 0.004 | 0.006 | 0.008 | 0.010 | 0.012 | 0.014 | 0.016 | 0.018 | 0.020 |
|  |  | Frequent | 0.006 | 0.010 | 0.013 | 0.015 | 0.018 | 0.020 | 0.023 | 0.025 | 0.027 | 0.030 |
|  | Females | No | 0.002 | 0.003 | 0.004 | 0.006 | 0.007 | 0.009 | 0.010 | 0.011 | 0.013 | 0.014 |
|  |  | Non-regular | 0.001 | 0.002 | 0.004 | 0.005 | 0.006 | 0.007 | 0.008 | 0.009 | 0.010 | 0.011 |
|  |  | Frequent | 0.005 | 0.007 | 0.009 | 0.011 | 0.013 | 0.015 | 0.017 | 0.018 | 0.020 | 0.022 |
| **Weight status** | Males | Normal | 0.003 | 0.005 | 0.008 | 0.011 | 0.014 | 0.016 | 0.019 | 0.022 | 0.024 | 0.027 |
|  |  | Underweight | 0.005 | 0.010 | 0.014 | 0.019 | 0.024 | 0.028 | 0.033 | 0.037 | 0.042 | 0.046 |
|  |  | Overweight | 0.002 | 0.005 | 0.007 | 0.010 | 0.013 | 0.016 | 0.018 | 0.021 | 0.024 | 0.026 |
|  |  | Obesity | 0.002 | 0.005 | 0.007 | 0.010 | 0.012 | 0.014 | 0.017 | 0.019 | 0.021 | 0.024 |
|  | Females | Normal | 0.002 | 0.003 | 0.004 | 0.006 | 0.007 | 0.009 | 0.010 | 0.011 | 0.013 | 0.014 |
|  |  | Underweight | 0.003 | 0.006 | 0.008 | 0.011 | 0.013 | 0.016 | 0.018 | 0.020 | 0.023 | 0.025 |
|  |  | Overweight | 0.001 | 0.002 | 0.004 | 0.005 | 0.006 | 0.007 | 0.009 | 0.010 | 0.011 | 0.012 |
|  |  | Obesity | 0.001 | 0.002 | 0.004 | 0.005 | 0.006 | 0.007 | 0.008 | 0.010 | 0.011 | 0.012 |

Model adjusted for age, education levels, residential regions, marital status, chronic disease conditions, social participation, and weight status.

DS: depressive symptoms.

**Table S17** Estimated transition probabilities from severe-DS to non-DS over 10 years, stratified by sex and other factors

|  | | | **Severe-DS → Non-DS** | | | | | | | | | |
| --- | --- | --- | --- | --- | --- | --- | --- | --- | --- | --- | --- | --- |
|  |  |  | Year 1 | Year 2 | Year 3 | Year 4 | Year 5 | Year 6 | Year 7 | Year 8 | Year 9 | Year 10 |
| **Age, years** | Males | 45-54 | 0.170 | 0.387 | 0.539 | 0.634 | 0.691 | 0.725 | 0.744 | 0.755 | 0.761 | 0.763 |
|  |  | 55-64 | 0.176 | 0.400 | 0.555 | 0.650 | 0.706 | 0.738 | 0.755 | 0.763 | 0.766 | 0.766 |
|  |  | ≥65 | 0.206 | 0.439 | 0.580 | 0.654 | 0.689 | 0.702 | 0.703 | 0.696 | 0.687 | 0.675 |
|  | Females | 45-54 | 0.129 | 0.307 | 0.441 | 0.532 | 0.590 | 0.627 | 0.651 | 0.666 | 0.675 | 0.680 |
|  |  | 55-64 | 0.135 | 0.321 | 0.461 | 0.555 | 0.615 | 0.653 | 0.677 | 0.691 | 0.700 | 0.704 |
|  |  | ≥65 | 0.161 | 0.362 | 0.501 | 0.587 | 0.636 | 0.664 | 0.678 | 0.684 | 0.685 | 0.683 |
| **Chronic disease conditions** | Males | 0 | 0.170 | 0.387 | 0.539 | 0.634 | 0.691 | 0.725 | 0.744 | 0.755 | 0.761 | 0.763 |
|  |  | 1-2 | 0.149 | 0.343 | 0.480 | 0.567 | 0.620 | 0.651 | 0.669 | 0.679 | 0.684 | 0.686 |
|  |  | ≥3 | 0.109 | 0.262 | 0.378 | 0.454 | 0.503 | 0.532 | 0.549 | 0.558 | 0.562 | 0.563 |
|  | Females | 0 | 0.129 | 0.307 | 0.441 | 0.532 | 0.590 | 0.627 | 0.651 | 0.666 | 0.675 | 0.680 |
|  |  | 1-2 | 0.113 | 0.268 | 0.386 | 0.466 | 0.517 | 0.550 | 0.570 | 0.583 | 0.591 | 0.595 |
|  |  | ≥3 | 0.082 | 0.201 | 0.295 | 0.361 | 0.405 | 0.433 | 0.451 | 0.463 | 0.469 | 0.473 |
| **Social participation** | Males | No | 0.170 | 0.387 | 0.539 | 0.634 | 0.691 | 0.725 | 0.744 | 0.755 | 0.761 | 0.763 |
|  |  | Non-regular | 0.158 | 0.369 | 0.522 | 0.622 | 0.683 | 0.721 | 0.744 | 0.757 | 0.765 | 0.769 |
|  |  | Frequent | 0.181 | 0.407 | 0.563 | 0.659 | 0.716 | 0.749 | 0.768 | 0.779 | 0.784 | 0.787 |
|  | Females | No | 0.129 | 0.307 | 0.441 | 0.532 | 0.590 | 0.627 | 0.651 | 0.666 | 0.675 | 0.680 |
|  |  | Non-regular | 0.120 | 0.292 | 0.426 | 0.519 | 0.580 | 0.621 | 0.647 | 0.664 | 0.675 | 0.682 |
|  |  | Frequent | 0.139 | 0.326 | 0.466 | 0.558 | 0.617 | 0.655 | 0.678 | 0.692 | 0.701 | 0.706 |
| **Weight status** | Males | Normal | 0.170 | 0.387 | 0.539 | 0.634 | 0.691 | 0.725 | 0.744 | 0.755 | 0.761 | 0.763 |
|  |  | Underweight | 0.173 | 0.384 | 0.524 | 0.608 | 0.656 | 0.683 | 0.697 | 0.704 | 0.706 | 0.706 |
|  |  | Overweight | 0.186 | 0.414 | 0.568 | 0.661 | 0.715 | 0.746 | 0.763 | 0.772 | 0.776 | 0.778 |
|  |  | Obesity | 0.176 | 0.399 | 0.553 | 0.649 | 0.707 | 0.740 | 0.760 | 0.771 | 0.777 | 0.779 |
|  | Females | Normal | 0.129 | 0.307 | 0.441 | 0.532 | 0.590 | 0.627 | 0.651 | 0.666 | 0.675 | 0.680 |
|  |  | Underweight | 0.131 | 0.303 | 0.426 | 0.505 | 0.554 | 0.583 | 0.601 | 0.611 | 0.617 | 0.620 |
|  |  | Overweight | 0.142 | 0.332 | 0.470 | 0.561 | 0.618 | 0.654 | 0.676 | 0.689 | 0.697 | 0.702 |
|  |  | Obesity | 0.134 | 0.318 | 0.456 | 0.549 | 0.609 | 0.647 | 0.671 | 0.686 | 0.695 | 0.701 |

Model adjusted for age, education levels, residential regions, marital status, chronic disease conditions, social participation, and weight status.

DS: depressive symptoms.

**Table S18** Estimated transition probabilities from severe-DS to mild-DS over 10 years, stratified by sex and other factors

|  | | | **Severe-DS → Mild-DS** | | | | | | | | | |
| --- | --- | --- | --- | --- | --- | --- | --- | --- | --- | --- | --- | --- |
|  |  |  | Year 1 | Year 2 | Year 3 | Year 4 | Year 5 | Year 6 | Year 7 | Year 8 | Year 9 | Year 10 |
| **Age, years** | Males | 45-54 | 0.323 | 0.301 | 0.246 | 0.205 | 0.178 | 0.162 | 0.151 | 0.145 | 0.141 | 0.139 |
|  |  | 55-64 | 0.309 | 0.283 | 0.227 | 0.185 | 0.158 | 0.142 | 0.132 | 0.125 | 0.121 | 0.119 |
|  |  | ≥65 | 0.338 | 0.279 | 0.208 | 0.161 | 0.134 | 0.118 | 0.108 | 0.101 | 0.097 | 0.094 |
|  | Females | 45-54 | 0.305 | 0.314 | 0.279 | 0.248 | 0.226 | 0.211 | 0.202 | 0.196 | 0.192 | 0.189 |
|  |  | 55-64 | 0.293 | 0.298 | 0.261 | 0.229 | 0.206 | 0.190 | 0.180 | 0.174 | 0.169 | 0.166 |
|  |  | ≥65 | 0.327 | 0.305 | 0.252 | 0.213 | 0.187 | 0.170 | 0.159 | 0.152 | 0.148 | 0.144 |
| **Chronic disease conditions** | Males | 0 | 0.323 | 0.301 | 0.246 | 0.205 | 0.178 | 0.162 | 0.151 | 0.145 | 0.141 | 0.139 |
|  |  | 1-2 | 0.312 | 0.305 | 0.263 | 0.228 | 0.206 | 0.191 | 0.182 | 0.176 | 0.172 | 0.169 |
|  |  | ≥3 | 0.281 | 0.305 | 0.284 | 0.261 | 0.244 | 0.232 | 0.224 | 0.218 | 0.213 | 0.210 |
|  | Females | 0 | 0.305 | 0.314 | 0.279 | 0.248 | 0.226 | 0.211 | 0.202 | 0.196 | 0.192 | 0.189 |
|  |  | 1-2 | 0.292 | 0.314 | 0.292 | 0.269 | 0.253 | 0.241 | 0.234 | 0.229 | 0.226 | 0.223 |
|  |  | ≥3 | 0.305 | 0.314 | 0.279 | 0.248 | 0.226 | 0.211 | 0.202 | 0.196 | 0.192 | 0.189 |
| **Social participation** | Males | No | 0.323 | 0.301 | 0.246 | 0.205 | 0.178 | 0.162 | 0.151 | 0.145 | 0.141 | 0.139 |
|  |  | Non-regular | 0.337 | 0.321 | 0.265 | 0.220 | 0.190 | 0.171 | 0.159 | 0.152 | 0.147 | 0.144 |
|  |  | Frequent | 0.362 | 0.324 | 0.255 | 0.206 | 0.175 | 0.156 | 0.144 | 0.137 | 0.133 | 0.130 |
|  | Females | No | 0.305 | 0.314 | 0.279 | 0.248 | 0.226 | 0.211 | 0.202 | 0.196 | 0.192 | 0.189 |
|  |  | Non-regular | 0.316 | 0.332 | 0.297 | 0.263 | 0.238 | 0.222 | 0.210 | 0.203 | 0.198 | 0.195 |
|  |  | Frequent | 0.344 | 0.341 | 0.293 | 0.252 | 0.225 | 0.207 | 0.195 | 0.187 | 0.182 | 0.179 |
| **Weight status** | Males | Normal | 0.323 | 0.301 | 0.246 | 0.205 | 0.178 | 0.162 | 0.151 | 0.145 | 0.141 | 0.139 |
|  |  | Underweight | 0.321 | 0.300 | 0.251 | 0.216 | 0.195 | 0.181 | 0.173 | 0.168 | 0.165 | 0.163 |
|  |  | Overweight | 0.351 | 0.312 | 0.247 | 0.201 | 0.172 | 0.155 | 0.145 | 0.139 | 0.135 | 0.133 |
|  |  | Obesity | 0.339 | 0.308 | 0.247 | 0.201 | 0.173 | 0.155 | 0.144 | 0.138 | 0.134 | 0.131 |
|  | Females | Normal | 0.305 | 0.314 | 0.279 | 0.248 | 0.226 | 0.211 | 0.202 | 0.196 | 0.192 | 0.189 |
|  |  | Underweight | 0.303 | 0.314 | 0.286 | 0.261 | 0.244 | 0.233 | 0.227 | 0.222 | 0.219 | 0.217 |
|  |  | Overweight | 0.334 | 0.330 | 0.285 | 0.248 | 0.223 | 0.207 | 0.197 | 0.190 | 0.186 | 0.183 |
|  |  | Obesity | 0.321 | 0.323 | 0.282 | 0.247 | 0.222 | 0.206 | 0.195 | 0.188 | 0.184 | 0.181 |

Model adjusted for age, education levels, residential regions, marital status, chronic disease conditions, social participation, and weight status.

DS: depressive symptoms.

**Table S19** Estimated probabilities of staying in severe-DS over 10 years, stratified by sex and other factors

|  | | | **Severe-DS → Severe-DS** | | | | | | | | | |
| --- | --- | --- | --- | --- | --- | --- | --- | --- | --- | --- | --- | --- |
|  |  |  | Year 1 | Year 2 | Year 3 | Year 4 | Year 5 | Year 6 | Year 7 | Year 8 | Year 9 | Year 10 |
| **Age, years** | Males | 45-54 | 0.503 | 0.305 | 0.205 | 0.148 | 0.115 | 0.095 | 0.083 | 0.075 | 0.071 | 0.068 |
|  |  | 55-64 | 0.507 | 0.304 | 0.200 | 0.140 | 0.106 | 0.085 | 0.072 | 0.065 | 0.060 | 0.057 |
|  |  | ≥65 | 0.431 | 0.238 | 0.148 | 0.101 | 0.075 | 0.060 | 0.052 | 0.046 | 0.043 | 0.041 |
|  | Females | 45-54 | 0.564 | 0.375 | 0.275 | 0.214 | 0.176 | 0.152 | 0.137 | 0.127 | 0.120 | 0.116 |
|  |  | 55-64 | 0.569 | 0.375 | 0.270 | 0.206 | 0.165 | 0.140 | 0.123 | 0.112 | 0.106 | 0.101 |
|  |  | ≥65 | 0.501 | 0.312 | 0.217 | 0.162 | 0.129 | 0.109 | 0.097 | 0.089 | 0.083 | 0.080 |
| **Chronic disease conditions** | Males | 0 | 0.503 | 0.305 | 0.205 | 0.148 | 0.115 | 0.095 | 0.083 | 0.075 | 0.071 | 0.068 |
|  |  | 1-2 | 0.531 | 0.338 | 0.239 | 0.181 | 0.146 | 0.125 | 0.112 | 0.103 | 0.098 | 0.095 |
|  |  | ≥3 | 0.595 | 0.407 | 0.303 | 0.241 | 0.201 | 0.176 | 0.159 | 0.148 | 0.141 | 0.136 |
|  | Females | 0 | 0.564 | 0.375 | 0.275 | 0.214 | 0.176 | 0.152 | 0.137 | 0.127 | 0.120 | 0.116 |
|  |  | 1-2 | 0.592 | 0.412 | 0.314 | 0.254 | 0.217 | 0.193 | 0.178 | 0.168 | 0.161 | 0.157 |
|  |  | ≥3 | 0.653 | 0.483 | 0.385 | 0.324 | 0.285 | 0.259 | 0.242 | 0.230 | 0.223 | 0.217 |
| **Social participation** | Males | No | 0.503 | 0.305 | 0.205 | 0.148 | 0.115 | 0.095 | 0.083 | 0.075 | 0.071 | 0.068 |
|  |  | Non-regular | 0.503 | 0.305 | 0.206 | 0.150 | 0.116 | 0.096 | 0.083 | 0.075 | 0.070 | 0.067 |
|  |  | Frequent | 0.454 | 0.262 | 0.172 | 0.122 | 0.094 | 0.077 | 0.067 | 0.061 | 0.057 | 0.055 |
|  | Females | No | 0.564 | 0.375 | 0.275 | 0.214 | 0.176 | 0.152 | 0.137 | 0.127 | 0.120 | 0.116 |
|  |  | Non-regular | 0.563 | 0.374 | 0.274 | 0.214 | 0.176 | 0.151 | 0.135 | 0.125 | 0.118 | 0.113 |
|  |  | Frequent | 0.516 | 0.329 | 0.235 | 0.181 | 0.147 | 0.126 | 0.112 | 0.104 | 0.098 | 0.095 |
| **Weight status** | Males | Normal | 0.503 | 0.305 | 0.205 | 0.148 | 0.115 | 0.095 | 0.083 | 0.075 | 0.071 | 0.068 |
|  |  | Underweight | 0.501 | 0.306 | 0.209 | 0.155 | 0.124 | 0.106 | 0.096 | 0.089 | 0.085 | 0.083 |
|  |  | Overweight | 0.458 | 0.265 | 0.174 | 0.124 | 0.096 | 0.079 | 0.069 | 0.064 | 0.060 | 0.058 |
|  |  | Obesity | 0.480 | 0.284 | 0.188 | 0.135 | 0.103 | 0.085 | 0.074 | 0.067 | 0.063 | 0.060 |
|  | Females | Normal | 0.564 | 0.375 | 0.275 | 0.214 | 0.176 | 0.152 | 0.137 | 0.127 | 0.120 | 0.116 |
|  |  | Underweight | 0.563 | 0.378 | 0.280 | 0.224 | 0.189 | 0.168 | 0.155 | 0.147 | 0.142 | 0.139 |
|  |  | Overweight | 0.522 | 0.334 | 0.239 | 0.185 | 0.151 | 0.130 | 0.117 | 0.109 | 0.104 | 0.101 |
|  |  | Obesity | 0.543 | 0.354 | 0.256 | 0.198 | 0.162 | 0.139 | 0.124 | 0.115 | 0.109 | 0.105 |

Model adjusted for age, education levels, residential regions, marital status, chronic disease conditions, social participation, and weight status.

DS: depressive symptoms.

**Table S20** Estimated transition probabilities from severe-DS to death over 10 years, stratified by sex and other factors

|  | | | **Severe-DS → Death** | | | | | | | | | |
| --- | --- | --- | --- | --- | --- | --- | --- | --- | --- | --- | --- | --- |
|  |  |  | Year 1 | Year 2 | Year 3 | Year 4 | Year 5 | Year 6 | Year 7 | Year 8 | Year 9 | Year 10 |
| **Age, years** | Males | 45-54 | 0.004 | 0.008 | 0.011 | 0.014 | 0.016 | 0.019 | 0.022 | 0.024 | 0.027 | 0.030 |
|  |  | 55-64 | 0.007 | 0.013 | 0.018 | 0.024 | 0.030 | 0.036 | 0.041 | 0.047 | 0.053 | 0.058 |
|  |  | ≥65 | 0.025 | 0.045 | 0.064 | 0.083 | 0.102 | 0.120 | 0.138 | 0.156 | 0.173 | 0.190 |
|  | Females | 45-54 | 0.002 | 0.003 | 0.005 | 0.006 | 0.008 | 0.009 | 0.011 | 0.012 | 0.013 | 0.015 |
|  |  | 55-64 | 0.003 | 0.006 | 0.008 | 0.011 | 0.014 | 0.017 | 0.020 | 0.023 | 0.025 | 0.028 |
|  |  | ≥65 | 0.011 | 0.020 | 0.029 | 0.039 | 0.048 | 0.057 | 0.066 | 0.075 | 0.084 | 0.093 |
| **Chronic disease conditions** | Males | 0 | 0.004 | 0.008 | 0.011 | 0.014 | 0.016 | 0.019 | 0.022 | 0.024 | 0.027 | 0.030 |
|  |  | 1-2 | 0.008 | 0.013 | 0.019 | 0.024 | 0.028 | 0.033 | 0.037 | 0.042 | 0.046 | 0.050 |
|  |  | ≥3 | 0.014 | 0.025 | 0.035 | 0.044 | 0.052 | 0.060 | 0.068 | 0.076 | 0.083 | 0.090 |
|  | Females | 0 | 0.002 | 0.003 | 0.005 | 0.006 | 0.008 | 0.009 | 0.011 | 0.012 | 0.013 | 0.015 |
|  |  | 1-2 | 0.003 | 0.006 | 0.009 | 0.011 | 0.013 | 0.016 | 0.018 | 0.020 | 0.023 | 0.025 |
|  |  | ≥3 | 0.006 | 0.011 | 0.015 | 0.019 | 0.024 | 0.028 | 0.031 | 0.035 | 0.039 | 0.043 |
| **Social participation** | Males | No | 0.004 | 0.008 | 0.011 | 0.014 | 0.016 | 0.019 | 0.022 | 0.024 | 0.027 | 0.030 |
|  |  | Non-regular | 0.002 | 0.004 | 0.006 | 0.009 | 0.010 | 0.012 | 0.014 | 0.016 | 0.018 | 0.020 |
|  |  | Frequent | 0.003 | 0.006 | 0.010 | 0.013 | 0.016 | 0.018 | 0.021 | 0.023 | 0.025 | 0.028 |
|  | Females | No | 0.002 | 0.003 | 0.005 | 0.006 | 0.008 | 0.009 | 0.011 | 0.012 | 0.013 | 0.015 |
|  |  | Non-regular | 0.001 | 0.002 | 0.003 | 0.004 | 0.005 | 0.006 | 0.007 | 0.008 | 0.009 | 0.011 |
|  |  | Frequent | 0.002 | 0.004 | 0.007 | 0.009 | 0.011 | 0.013 | 0.015 | 0.017 | 0.018 | 0.020 |
| **Weight status** | Males | Normal | 0.503 | 0.305 | 0.205 | 0.148 | 0.115 | 0.095 | 0.083 | 0.075 | 0.071 | 0.068 |
|  |  | Underweight | 0.005 | 0.010 | 0.015 | 0.020 | 0.025 | 0.029 | 0.034 | 0.038 | 0.043 | 0.047 |
|  |  | Overweight | 0.005 | 0.009 | 0.012 | 0.014 | 0.017 | 0.020 | 0.023 | 0.025 | 0.028 | 0.031 |
|  |  | Obesity | 0.005 | 0.009 | 0.012 | 0.015 | 0.017 | 0.020 | 0.022 | 0.024 | 0.027 | 0.029 |
|  | Females | Normal | 0.002 | 0.003 | 0.005 | 0.006 | 0.008 | 0.009 | 0.011 | 0.012 | 0.013 | 0.015 |
|  |  | Underweight | 0.002 | 0.005 | 0.008 | 0.010 | 0.013 | 0.015 | 0.017 | 0.020 | 0.022 | 0.025 |
|  |  | Overweight | 0.002 | 0.004 | 0.005 | 0.006 | 0.008 | 0.009 | 0.010 | 0.012 | 0.013 | 0.014 |
|  |  | Obesity | 0.002 | 0.004 | 0.005 | 0.007 | 0.008 | 0.009 | 0.010 | 0.012 | 0.013 | 0.014 |

Model adjusted for age, education levels, residential regions, marital status, chronic disease conditions, social participation, and weight status.

DS: depressive symptoms.

**

**

**Figure S3 Probability curves of transitioning to death over 10 years, stratified by sex, age, chronic diseases conditions, social participation and weight status.** Model adjusted for age, education levels, residential regions, marital status, chronic disease conditions, social participation, and weight status. DS: depressive symptoms.





**Figure S4 Probability curves of recovering over 10 years, stratified by sex, age, chronic diseases conditions, social participation and weight status.** Model adjusted for age, education levels, residential regions, marital status, chronic disease conditions, social participation, and weight status. DS: depressive symptoms.





**Figure S5 Probability curves of staying in original states over 10 years, stratified by sex, age, chronic diseases conditions, social participation and weight status.** Model adjusted for age, education levels, residential regions, marital status, chronic disease conditions, social participation, and weight status. DS: depressive symptoms.

**Table S21** Estimated total length of stay in 10 years stratified by sex and other factors

|  |  | **Males (years)** | | | | **Females (years)** | | | |
| --- | --- | --- | --- | --- | --- | --- | --- | --- | --- |
|  |  | **Non-DS** | **Mild-DS** | **Severe-DS** | **Death** | **Non-DS** | **Mild-DS** | **Severe-DS** | **Death** |
| **Age, years** | 45-54 | 8.196 | 1.190 | 0.483 | 0.131 | 7.564 | 1.589 | 0.782 | 0.065 |
|  | 55-64 | 8.268 | 1.032 | 0.407 | 0.293 | 7.778 | 1.408 | 0.673 | 0.141 |
|  | ≥65 | 7.812 | 0.881 | 0.331 | 0.976 | 7.686 | 1.259 | 0.583 | 0.472 |
| **Chronic disease conditions** | 0 | 8.196 | 1.190 | 0.483 | 0.131 | 7.564 | 1.589 | 0.782 | 0.065 |
|  | 1-2 | 7.627 | 1.479 | 0.680 | 0.214 | 6.891 | 1.926 | 1.076 | 0.1.7 |
|  | ≥3 | 6.770 | 1.900 | 0.960 | 0.370 | 5.951 | 2.398 | 1.471 | 0.180 |
| **Social participation** | No | 8.196 | 1.190 | 0.483 | 0.131 | 7.564 | 1.589 | 0.782 | 0.065 |
|  | Non-regular | 8.248 | 1.198 | 0.457 | 0.097 | 7.618 | 1.596 | 0.736 | 0.049 |
|  | Frequent | 8.396 | 1.101 | 0.396 | 0.108 | 7.802 | 1.480 | 0.646 | 0.072 |
| **Weight status** | Underweight | 7.704 | 1.446 | 0.621 | 0.229 | 6.988 | 1.901 | 0.992 | 0.118 |
|  | Normal | 8.196 | 1.190 | 0.483 | 0.131 | 7.564 | 1.589 | 0.782 | 0.065 |
|  | Overweight | 8.301 | 1.141 | 0.425 | 0.133 | 7.708 | 1.534 | 0.696 | 0.062 |
|  | Obesity | 8.339 | 1.119 | 0.432 | 0.111 | 7.739 | 1.502 | 0.705 | 0.054 |

Model adjusted for age, education levels, residential regions, marital status, chronic disease conditions, social participation, and weight status.

DS: depressive symptoms.

**Table S22** Estimated mean sojourn time stratified by sex and other factors

|  | |  | **Males** | | | **Females** | | |
| --- | --- | --- | --- | --- | --- | --- | --- | --- |
|  | |  | **Mean (±SE) (years)** | | | **Mean (±SE) (years)** | | |
|  | |  | **Non-DS** | **Mild-DS** | **Severe-DS** | **Non-DS** | **Mild-DS** | **Severe-DS** |
| **Age, years** | 45-54 | | 6.926±0.462 | 0.837±0.048 | 1.214±0.126 | 5.419±0.332 | 0.911±0.049 | 1.440±0.138 |
|  | 55-64 | | 7.374±0.460 | 0.808±0.043 | 1.248±0.124 | 5.874±0.349 | 0.885±0.045 | 1.484±0.138 |
|  | ≥65 | | 7.100±0.432 | 0.754±0.042 | 0.974±0.101 | 6.095±0.372 | 0.821±0.046 | 1.172±0.117 |
| **Chronic disease conditions** | 0 | | 6.926±0.462 | 0.837±0.048 | 1.214±0.126 | 5.419±0.332 | 0.911±0.049 | 1.440±0.138 |
|  | 1-2 | | 5.371±0.347 | 0.852±0.047 | 1.307±0.124 | 4.215±0.247 | 0.922±0.047 | 1.556±0.132 |
|  | ≥3 | | 4.057±0.286 | 0.959±0.057 | 1.624±0.157 | 3.200±0.210 | 1.035±0.057 | 1.592±0.171 |
| **Social participation** | No | | 6.926±0.462 | 0.837±0.048 | 1.214±0.126 | 5.419±0.332 | 0.911±0.049 | 1.440±0.138 |
|  | Non-regular | | 7.557±0.534 | 0.915±0.056 | 1.221±0.137 | 5.899±0.390 | 0.994±0.058 | 1.447±0.152 |
|  | Frequent | | 8.196±0.573 | 0.872±0.053 | 1.044±0.120 | 6.388±0.405 | 0.946±0.055 | 1.235±0.131 |
| **Weight status** | Underweight | | 5.147±0.530 | 0.796±0.066 | 1.196±0.174 | 4.041±0.406 | 0.866±0.071 | 1.420±0.197 |
|  | Normal | | 6.926±0.462 | 0.837±0.048 | 1.214±0.126 | 5.419±0.332 | 0.911±0.049 | 1.440±0.138 |
|  | Overweight | | 7.431±0.520 | 0.837±0.051 | 1.056±0.117 | 5.823±0.369 | 0.909±0.051 | 1.254±0.124 |
|  | Obesity | | 7.674±0.613 | 0.848±0.058 | 1.131±0.140 | 5.998±0.441 | 0.921±0.059 | 1.343±0.152 |

Model adjusted for age, education levels, residential regions, marital status, chronic disease conditions, social participation, and weight status.

SE: standard errors.

DS: depressive symptoms.

**Table S23** Baseline characteristics of participants in the sensitivity analysis

| **Factors** | **Male** | **Female** |
| --- | --- | --- |
|  | (n=7149) | (n=6000) |
| **Age (years)** |  |  |
| 45-54 | 3167 (44.3) | 2879 (48.0) |
| 55-64 | 2458 (34.4) | 1949 (32.5) |
| ≥65 | 1524 (21.3) | 1172 (19.5) |
| **Education levels** |  |  |
| Elementary below | 1776 (24.8) | 2889 (48.2) |
| Elementary | 2267 (31.7) | 1397 (23.3) |
| Middle school and above | 3106 (43.4) | 1714 (28.6) |
| **Marital status** |  |  |
| Married | 6706 (93.8) | 5321 (88.7) |
| Others | 443 (6.2) | 679 (11.3) |
| **Residential regions** |  |  |
| Urban | 3108 (43.5) | 2844 (47.4) |
| Rural | 4041 (56.5) | 3156 (52.6) |
| **Chronic disease conditions** |  |  |
| 0 | 3288 (46.0) | 2663 (44.4) |
| 1-2 | 3084 (43.1) | 2599 (43.3) |
| ≥3 | 777 (10.9) | 738 (12.3) |
| **Social participation** |  |  |
| No | 3079 (43.1) | 2695 (44.9) |
| Non-regular | 1758 (24.6) | 1204 (20.1) |
| Frequent | 2312 (32.3) | 2101 (35.0) |
| **Weight status** |  |  |
| Underweight | 310 (4.3) | 242 (4.0) |
| Normal | 3887 (54.4) | 2724 (45.4) |
| Overweight | 2250 (31.5) | 2125 (35.4) |
| Obesity | 702 (9.8) | 910 (15.2) |
| **CESD-10 score** | 5.2±4.5 | 6.1±4.9 |

Values were n (percentages) or mean±standard deviation.

CESD-10: the 10-item Center for Epidemiological Studies Depression Scale.

**Table S24** Observed frequencies of transitions between DS states in the sensitivity analysis

| **Original state** | **Follow-up state** | | | |
| --- | --- | --- | --- | --- |
|  | **Non-DS** | **Mild-DS** | **Severe-DS** | **Death** |
| **Overall** |  |  |  |  |
| Non-DS | 26902 (81.8) | 4015 (12.2) | 1257 (3.8) | 696 (2.1) |
| Mild-DS | 2536 (62.9) | 968 (24.0) | 368 (9.1) | 161 (4.0) |
| Severe-DS | 724 (45.05) | 478 (29.7) | 314 (19.5) | 91 (5.7) |
| **Males** |  |  |  |  |
| Non-DS | 15430 (84.1) | 1898 (10.4) | 523 (2.9) | 489 (2.7) |
| Mild-DS | 1254 (67.5) | 397 (21.4) | 114 (6.1) | 94 (5.1) |
| Severe-DS | 301 (48.9) | 154 (25.0) | 105 (17.1) | 56 (9.1) |
| **Females** |  |  |  |  |
| Non-DS | 11472 (789.0) | 2117 (14.6) | 734 (5.1) | 207 (1.4) |
| Mild-DS | 1282 (59.0) | 571 (26.3) | 254 (11.7) | 67 (3.1) |
| Severe-DS | 423 (42.7) | 324 (32.7) | 209 (21.1) | 35 (3.5) |

Values were n ((percentages).

DS: depressive symptoms.

**Table S25** Estimated transition intensities in the sensitivity analysis

| **Original state** | **Follow-up state** | | | |
| --- | --- | --- | --- | --- |
|  | **Non-DS** | **Mild-DS** | **Severe-DS** | **Death** |
| **Overall** |  |  |  |  |
| Non-DS | -0.167  (-0.175, -0.159) | 0.160  (0.153,0.168) | 0 | 0.006  (0.005,0.008) |
| Mild-DS | 0.793  (0.749,0.839) | -1.236  (-1.297, -1.178) | 0.420  (0.374,0.471) | 0.024  (0.015,0.036) |
| Severe-DS | 0 | 1.065  (0.945,1.199) | -1.101  (-1.237, -0.980) | 0.036  (0.023,0.057) |
| **Males** |  |  |  |  |
| Non-DS | -0.151  (-0.162, -0.140) | 0.142  (0.131,0.154) | 0 | 0.009  (0.007,0.010) |
| Mild-DS | 0.894  (0.819,0.976) | -1.293  (-1.387, -1.204) | 0.374  (0.312,0.448) | 0.025  (0.012,0.050) |
| Severe-DS | 0 | 1.090  (0.906,1.312) | -1.164  (-1.393, -0.974) | 0.074  (0.047,0.117) |
| **Females** |  |  |  |  |
| Non-DS | -0.193  (-0.206, -0.181) | 0.190  (0.178,0.203) | 0 | 0.003  (0.002,0.005) |
| Mild-DS | 0.738  (0.683,0.798) | -1.239  (-1.327, -1.156) | 0.478  (0.407,0.561) | 0.022  (0.013,0.039) |
| Severe-DS | 0 | 1.099  (0.931,1.297) | -1.116  (-1.315, -0.947) | 0.017  (0.006, 0.048) |

Values were intensity (95%CI).

DS: depressive symptoms.

**Table S26** Estimated transition probabilities over 10 years for males in the sensitivity analysis

| **Years** | **Non-DS→**  **Non-DS** | **Non-DS→**  **Mild-DS** | **Non-DS→**  **Severe-DS** | **Non-DS→**  **Death** | **Mild-DS→**  **Non-DS** | **Mild-DS→**  **Mild-DS** |
| --- | --- | --- | --- | --- | --- | --- |
| Year 1 | 0.900 | 0.078 | 0.012 | 0.010 | 0.492 | 0.364 |
| Year 2 | 0.851 | 0.103 | 0.025 | 0.021 | 0.648 | 0.213 |
| Year 3 | 0.822 | 0.113 | 0.032 | 0.033 | 0.709 | 0.161 |
| Year 4 | 0.803 | 0.117 | 0.035 | 0.045 | 0.733 | 0.138 |
| Year 5 | 0.788 | 0.118 | 0.037 | 0.058 | 0.739 | 0.127 |
| Year 6 | 0.775 | 0.117 | 0.038 | 0.070 | 0.738 | 0.120 |
| Year 7 | 0.764 | 0.116 | 0.038 | 0.082 | 0.732 | 0.116 |
| Year 8 | 0.753 | 0.115 | 0.037 | 0.094 | 0.725 | 0.113 |
| Year 9 | 0.743 | 0.113 | 0.037 | 0.106 | 0.716 | 0.111 |
| Year 10 | 0.733 | 0.112 | 0.037 | 0.118 | 0.707 | 0.109 |

**Table S26 (cont.)** Estimated transition probabilities over 10 years for males in the sensitivity analysis

| **Years** | **Mild-DS→**  **Severe-DS** | **Mild-DS→**  **Death** | **Severe-DS→**  **Non-DS** | **Severe-DS→**  **Mild-DS** | **Severe-DS→**  **Severe-DS** | **Severe-DS→**  **Death** |
| --- | --- | --- | --- | --- | --- | --- |
| Year 1 | 0.121 | 0.024 | 0.220 | 0.351 | 0.375 | 0.054 |
| Year 2 | 0.095 | 0.044 | 0.453 | 0.277 | 0.186 | 0.084 |
| Year 3 | 0.069 | 0.061 | 0.585 | 0.201 | 0.108 | 0.106 |
| Year 4 | 0.054 | 0.075 | 0.649 | 0.157 | 0.072 | 0.122 |
| Year 5 | 0.046 | 0.088 | 0.678 | 0.133 | 0.054 | 0.136 |
| Year 6 | 0.041 | 0.101 | 0.687 | 0.120 | 0.044 | 0.148 |
| Year 7 | 0.039 | 0.113 | 0.687 | 0.113 | 0.039 | 0.160 |
| Year 8 | 0.037 | 0.125 | 0.683 | 0.109 | 0.037 | 0.172 |
| Year 9 | 0.036 | 0.137 | 0.676 | 0.106 | 0.035 | 0.183 |
| Year 10 | 0.036 | 0.148 | 0.669 | 0.104 | 0.034 | 0.194 |

DS: depressive symptoms.

**Table S27** Estimated transition probabilities over 10 years for females in the sensitivity analysis

| **Years** | **Non-DS→**  **Non-DS** | **Non-DS→**  **Mild-DS** | **Non-DS→**  **Severe-DS** | **Non-DS→**  **Death** | **Mild-DS→**  **Non-DS** | **Mild-DS→**  **Mild-DS** |
| --- | --- | --- | --- | --- | --- | --- |
| Year 1 | 0.868 | 0.106 | 0.021 | 0.004 | 0.413 | 0.405 |
| Year 2 | 0.801 | 0.143 | 0.045 | 0.011 | 0.556 | 0.271 |
| Year 3 | 0.763 | 0.160 | 0.059 | 0.017 | 0.622 | 0.224 |
| Year 4 | 0.740 | 0.168 | 0.067 | 0.024 | 0.654 | 0.201 |
| Year 5 | 0.725 | 0.172 | 0.071 | 0.032 | 0.669 | 0.188 |
| Year 6 | 0.714 | 0.174 | 0.073 | 0.039 | 0.675 | 0.181 |
| Year 7 | 0.705 | 0.174 | 0.074 | 0.047 | 0.676 | 0.176 |
| Year 8 | 0.698 | 0.174 | 0.074 | 0.054 | 0.675 | 0.173 |
| Year 9 | 0.692 | 0.173 | 0.074 | 0.061 | 0.671 | 0.170 |
| Year 10 | 0.686 | 0.171 | 0.074 | 0.069 | 0.667 | 0.169 |

**Table 27 (cont.)** Estimated transition probabilities over 10 years for females in the sensitivity analysis

| **Years** | **Mild-DS→**  **Severe-DS** | **Mild-DS→**  **Death** | **Severe-DS→**  **Non-DS** | **Severe-DS→**  **Mild-DS** | **Severe-DS→**  **Severe-DS** | **Severe-DS→**  **Death** |
| --- | --- | --- | --- | --- | --- | --- |
| Year 1 | 0.165 | 0.017 | 0.188 | 0.380 | 0.414 | 0.017 |
| Year 2 | 0.144 | 0.028 | 0.398 | 0.332 | 0.239 | 0.031 |
| Year 3 | 0.116 | 0.038 | 0.527 | 0.267 | 0.162 | 0.043 |
| Year 4 | 0.098 | 0.047 | 0.598 | 0.226 | 0.123 | 0.053 |
| Year 5 | 0.088 | 0.055 | 0.636 | 0.202 | 0.101 | 0.062 |
| Year 6 | 0.082 | 0.062 | 0.654 | 0.188 | 0.088 | 0.070 |
| Year 7 | 0.078 | 0.070 | 0.662 | 0.179 | 0.081 | 0.077 |
| Year 8 | 0.076 | 0.077 | 0.664 | 0.174 | 0.077 | 0.085 |
| Year 9 | 0.074 | 0.084 | 0.663 | 0.170 | 0.075 | 0.092 |
| Year 10 | 0.073 | 0.092 | 0.660 | 0.168 | 0.073 | 0.099 |

DS: depressive symptoms.

**Table S28** Percentage of total length of stay over 10 years in the sensitivity analysis

| **Years** | **Males (%)** | | | | **Females (%)** | | | |
| --- | --- | --- | --- | --- | --- | --- | --- | --- |
|  | **Non-DS** | **Mild-DS** | **Severe-DS** | **Death** | **Non-DS** | **Mild-DS** | **Severe-DS** | **Death** |
| Year 1 | 94.3 | 4.7 | 0.5 | 0.5 | 92.6 | 6.4 | 0.9 | 0.2 |
| Year 2 | 90.8 | 7.0 | 1.2 | 1.0 | 87.9 | 9.6 | 2.1 | 0.5 |
| Year 3 | 88.4 | 8.3 | 1.8 | 1.6 | 84.6 | 11.5 | 3.2 | 0.8 |
| Year 4 | 86.6 | 9.1 | 2.2 | 2.1 | 82.2 | 12.7 | 4.0 | 1.1 |
| Year 5 | 85.2 | 9.6 | 2.5 | 2.7 | 80.4 | 13.6 | 4.5 | 1.5 |
| Year 6 | 84.0 | 10.0 | 2.7 | 3.3 | 79.0 | 14.2 | 5.0 | 1.8 |
| Year 7 | 83.0 | 10.2 | 2.8 | 4.0 | 77.9 | 14.7 | 5.3 | 2.2 |
| Year 8 | 82.1 | 10.4 | 2.9 | 4.6 | 76.9 | 15.0 | 5.6 | 2.5 |
| Year 9 | 81.3 | 10.5 | 3.0 | 5.2 | 76.1 | 15.3 | 5.8 | 2.9 |
| Year 10 | 80.5 | 10.6 | 3.1 | 5.8 | 75.4 | 15.5 | 6.0 | 3.2 |

DS: depressive symptoms.

**Table S29** Estimated mean sojourn time in each DS state from the sensitivity analysis

| **DS state** | **Estimated mean sojourn time (years)** | **Standard errors** | **95% CI** | |  |
| --- | --- | --- | --- | --- | --- |
|  |  |  | **Lower** | **Upper** |  |
| **Overall** |  |  |  |  | |
| Non-DS | 5.961 | 0.150 | 5.674 | 6.264 | |
| Mild-DS | 0.793 | 0.020 | 0.754 | 0.833 | |
| Severe-DS | 0.881 | 0.056 | 0.778 | 0.997 | |
| **Males** |  |  |  |  | |
| Non-DS | 6.638 | 0.244 | 6.177 | 7.134 |  |
| Mild-DS | 0.773 | 0.028 | 0.720 | 0.831 |  |
| Severe-DS | 0.856 | 0.079 | 0.715 | 1.024 |  |
| **Females** |  |  |  |  | |
| Non-DS | 5.180 | 0.169 | 4.860 | 5.522 |  |
| Mild-DS | 0.807 | 0.028 | 0.753 | 0.865 |  |
| Severe-DS | 0.894 | 0.075 | 0.759 | 1.054 |  |

DS: depressive symptoms.

**Table S30** Hazard ratios of covariates associated with transitions between DS states in the sensitivity analysis

| **Factors** | **Deteriorate transition** | | | **Recovery transition** | | | **Death transition** | | |
| --- | --- | --- | --- | --- | --- | --- | --- | --- | --- |
|  | Non-DS→  Mild-DS | Mild-DS→  Severe-DS | Mild-DS→  Non-DS | | Severe-DS→  Mild-DS | Non-DS→  Death | | Mild-DS→  Death | Severe-DS→  Death |
| **Sex** |  |  |  | |  |  | |  |  |
| Males | 1 (ref) | 1 (ref) | 1 (ref) | | 1 (ref) | 1 (ref) | | 1 (ref) | 1 (ref) |
| Females | **1.193 (1.060,1.343)** | 1.261 (0.971,1.637) | **0.808 (0.705,0.925)** | | 1.065 (0.815,1.392) | **0.389 (0.270,0.560)** | | 0.753 (0.438,1.293) | **0.244 (0.097,0.612)** |
| **Age, years** |  |  |  | |  |  | |  |  |
| 45-54 | 1 (ref) | 1 (ref) | 1 (ref) | | 1 (ref) | 1 (ref) | | 1 (ref) | 1 (ref) |
| 55-64 | **0.874 (0.765,0.999)** | 0.896 (0.651,1.233) | 1.069 (0.915,1.250) | | 0.897 (0.642,1.254) | **2.262 (1.231,4.118)** | | 2.137 (0.783,8.833) | 1.937 (0.537,6.994) |
| ≥65 | **0.776 (0.667,0.902)** | 1.243 (0.849,1.820) | 1.031 (0.866,1.228) | | **6.720 (2.695,16.757)** | **8.492 (4.876,14.788)** | | **6.720 (2.695,16.757)** | **4.862 (1.435,16.475)** |
| **Education levels** |  |  |  | |  |  | |  |  |
| Elementary below | 1 (ref) | 1 (ref) | 1 (ref) | | 1 (ref) | 1 (ref) | | 1 (ref) | 1 (ref) |
| Elementary | **0.839 (0.720,0.978)** | 0.829 (0.600,1.145) | 1.118 (0.939,1.332) | | 1.029 (0.737,1.435) | 0.961 (0.688,1.341) | | 0.086 (0.002,4.417) | 2.235 (0.984,5.075) |
| Middle school and above | **0.610 (0.532,0.698)** | 0.722 (0.518,1.004) | 0.941 (0.805,1.099) | | 1.113 (0.795,1.558) | 0.755 (0.528,1.080) | | 0.799 (0.425,1.504) | 0.445 (0.081,2.454) |
| **Marital status** |  |  |  | |  |  | |  |  |
| Married | 1 (ref) | 1 (ref) | 1 (ref) | | 1 (ref) | 1 (ref) | | 1 (ref) | 1 (ref) |
| Others | 1.090 (0.928,1.280) | **2.088 (1.525,2.857)** | 0.906 (0.756,1.085) | | **0.684 (0.491,0.953)** | **2.088 (1.525,2.857)** | | **1.657 (1.004,2.737)** | 1.456 (0.677,3.128) |
| **Residential regions** |  |  |  | |  |  | |  |  |
| Urban | 1 (ref) | 1 (ref) | 1 (ref) | | 1 (ref) | 1 (ref) | | 1 (ref) | 1 (ref) |
| Rural | **1.410 (1.260,1.576)** | 1.081 (0.818,1.428) | 1.086 (0.955,1.236) | | 0.963 (0.722,1.285) | 1.246 (0.929,1.671) | | **0.679 (0.403,1.145)** | 0.668 (0.351,1.272) |
| **Chronic disease conditions** | |  |  | |  |  | |  |  |
| 0 | 1(ref) | 1(ref) | 1(ref) | | 1(ref) | 1(ref) | | 1(ref) | 1(ref) |
| 1-2 | **1.352 (1.183,1.545)** | 0.926 (0.639,1.343) | 1.069 (0.911,1.256) | | 0.837 (0.558,1.256) | **2.106 (1.329,3.336)** | | 0.838 (0.497,1.412) | 0.868 (0.343,2.200) |
| ≥3 | **1.632 (1.406,1.895)** | 0.813 (0.546,1.212) | 0.883 (0.743,1.049) | | 0.783 (0.514,1.191) | **4.266 (2.678,6.794)** | | 0.763 (0.393,1.482) | 1.084 (0.422,2.785) |
| **Social participation** |  |  |  | |  |  | |  |  |
| No | 1 (ref) | 1 (ref) | 1 (ref) | | 1 (ref) | 1 (ref) | | 1 (ref) | 1 (ref) |
| Non-regular | **0.844 (0.737,0.967)** | 0.856 (0.631,1.161) | **0.794 (0.679,0.928)** | | 0.867 (0.632,1.190) | 0.798 (0.581,1.096) | | 0.735 (0.364,1.484) | **0.342 (0.117,0.994)** |
| Frequent | **0.786 (0.692,0.892)** | 1.144 (0.825,1.586) | 0.876 (0.757,1.014) | | 1.212 (0.865,1.699) | **0.646 (0.466,0.894)** | | 1.162 (0.670,2.017) | 0.440 (0.168,1.154) |
| **Weight status** |  |  |  | |  |  | |  |  |
| Normal | 1 (ref) | 1 (ref) | 1 (ref) | | 1 (ref) | 1 (ref) | | 1 (ref) | 1 (ref) |
| Underweight | **1.671 (1.109,2.517)** | 0.800 (0.487,1.315) | 1.392 (0.863,2.244) | | 0.685 (0.404,1.160) | 0.858 (0.282,2.614) | | **3.611 (1.944,6.705)** | 0.803 (0.160,4.043) |
| Overweight | 0.959 (0.850,1.082) | 1.094 (0.794,1.506) | 1.041 (0.906,1.196) | | 1.174 (0.842,1.638) | 0.993 (0.739,1.332) | | 0.614 (0.288,1.310) | 1.806 (0.829,3.938) |
| Obesity | 0.872 (0.737,1.032) | 0.741 (0.520,1.055) | 0.991 (0.817,1.203) | | 0.818 (0.570,1.175) | 0.802 (0.524,1.227) | | 0.246 (0.015,4.045) | 1.651 (0.632,4.317) |

Values were hazard ratios (95% CI).

Boldfaced data indicate statistical signiﬁcance (P < 0.05).

DS: depressive symptoms.





**Figure S6** **Transition probability curves and percentage of total length of stay over 10 years** **in the sensitivity analysis.** (A)probability curves of progression to depressive states and transition to death; (B) probability curves of reversion; (C) probability curves of staying in an original state; (D) percentage of total length of stay. DS: depressive symptoms.





**Figure S7 Probability curves of worsening to depressive states over 10 years in the sensitivity analysis, stratified by sex, age, chronic diseases conditions, social participation and weight status.** Model adjusted for age, education levels, residential regions, marital status, chronic disease conditions, social participation, and weight status. DS: depressive symptoms.





**Figure S8 Probability curves of transitioning to death over 10 years in the sensitivity analysis, stratified by sex, age, chronic diseases conditions, social participation and weight status.** Model adjusted for age, education levels, residential regions, marital status, chronic disease conditions, social participation, and weight status. DS: depressive symptoms.





**Figure S9** **Probability curves of recovering over 10 years in the sensitivity analysis, stratified by sex, age, chronic diseases conditions, social participation and weight status.** Model adjusted for age, education levels, residential regions, marital status, chronic disease conditions, social participation, and weight status. DS: depressive symptoms.





**Figure S10 Probability curves of staying in original states over 10 years in the sensitivity analysis, stratified by sex, age, chronic diseases conditions, social participation and weight status.** Model adjusted for age, education levels, residential regions, marital status, chronic disease conditions, social participation, and weight status.  DS: depressive symptoms.





**Figure S11 Total length of stay in 10 years and mean sojourn time in the sensitivity analysis, stratified by sex, age, chronic diseases conditions, social participation and weight status.** (A) total length of stay in each state; (B)mean sojourn time in each state. Model adjusted for age, education levels, residential regions, marital status, chronic disease conditions, social participation, and weight status. DS: depressive symptoms.
